# A Jurassic stem pleurodire sheds light on the functional origin of neck retraction in turtles

**DOI:** 10.1038/srep42376

**Published:** 2017-02-16

**Authors:** Jérémy Anquetin, Haiyan Tong, Julien Claude

**Affiliations:** 1 JURASSICA Museum, Porrentruy, Switzerland; 2 Department of Geosciences, University of Fribourg, Fribourg, Switzerland; 3 Palaeontological Research and Education Centre, Mahasarakham University, Mahasarakham, Thailand; 4 Institut des Sciences de l’Evolution de Montpellier, UMR 5554 CNRS/UM/IRD/EPHE, Montpellier, France

## Abstract

Modern turtles are composed of two monophyletic groups, notably diagnosed by divergent neck retraction mechanisms. Pleurodires (side-necked turtles) bend their neck sideways and protect their head under the anterior margin of the carapace. Cryptodires (hidden-necked turtles) withdraw their neck and head in the vertical plane between the shoulder girdles. These two mechanisms of neck retraction appeared independently in the two lineages and are usually assumed to have evolved for protective reasons. Here we describe the neck of *Platychelys oberndorferi*, a Late Jurassic early stem pleurodire, and find remarkable convergent morphological and functional similarities with modern cryptodires. Partial vertical neck retraction in this taxon is interpreted to have enabled fast forward projection of the head during underwater prey capture and offers a likely explanation to the functional origin of neck retraction in modern cryptodires. Complete head withdrawal for protection may therefore have resulted from an exaptation in that group.

For supposed protective reasons, turtles developed complex double-bend neck mechanisms allowing neck and head retraction within the shell^1^. Living pleurodires (side-necked turtles) bend their neck sideways and tuck the head under the carapace anterior to the pectoral girdle. In contrast, living cryptodires (hidden-necked turtles) fold their neck in the vertical plane and withdraw the neck and head within the shell between the shoulder girdles. Pleurodires tend to have narrow cervical vertebrae with closely set and horizontal zygapophyses, whereas cryptodires usually have broader cervicals with widely spaced zygapophyses (usually vertically oriented on the posterior part of the neck) and often develop broad and more commonly double central articulations on posterior cervical vertebrae, a condition called ginglymoidy^2^,^3^. The pleurodiran condition clearly favors lateral movements of the neck, whereas the cryptodiran morphology promotes vertical movements and limits lateral bending in the posterior part of the neck.

The two mechanisms of neck retraction known in modern turtles evolved independently in the two lineages after the Late Jurassic from a supposed ancestral condition, called lateral head tucking, that did not allow full head retraction^4^. Although the fossil record is relatively poor for that matter, many Jurassic and Early Cretaceous stem cryptodires are known to have a relatively “primitive” cervical morphology characterized by a short, amphicoelous centrum and a relatively high neural arch^5^,^6^,^7^,^8^,^9^. In the present study, we describe the sixth and eighth cervical vertebrae of *Platychelys oberndorferi* Wagner, 1853^10^, an early stem pleurodire known from the Late Jurassic of Germany and Switzerland. These vertebrae show remarkable morphological and functional similarities with that of modern cryptodires, and reveal that *P. oberndorferi* was able to bend the neck in the vertical plane and partially withdraw the neck within the shell. Therefore, the typical cryptodiran neck retraction mechanism actually first evolved in Late Jurassic stem pleurodires and was only subsequently developed in cryptodires by convergence. Incidentally, *Xinjiangchelys qiguensis*, a Late Jurassic stem cryptodire from China^11^, seems to show a rather opposite trend with elongated cervical vertebrae reminiscent of those of modern pleurodires^4^,^12^. Early crown group turtles apparently experimented diverse types of neck retraction before the two mechanisms seen in modern turtles were fixed. Several anatomical features suggest that *P. oberndorferi* was an ambush predator practising ram (the body or the head moves toward the prey) or suction (the prey is sucked into the mouth) feeding, strongly reminding the behavior of the extant matamata (*Chelus fimbriatus*) and snapping turtles (*Chelydra serpentina* and *Macrochelys temminckii*). Since head and neck retraction was only partial in *P. oberndorferi*, we suggest that vertical neck retraction primarily evolved in this taxon to enable fast forward projection of the head and improve capture of darting prey. These results have important implications for the origin of neck retraction mechanisms in modern cryptodires.

## Results

**Systematic paleontology.**

TESTUDINES Batsch, 1788^13^.

PAN-PLEURODIRA Joyce *et al*.^14^.

PLATYCHELYIDAE Bräm, 1965^5^.

***Platychelys oberndorferi***. Wagner, 1853^10^.

**Type material.** A nearly complete carapace^10^,^15^, now lost^16^.

**Type horizon and locality**. Kelheim, Bavaria, Germany. Solnhofen Limestone Formation, Tithonian, Late Jurassic.

**Referred specimens**. See hypodigm of Cadena and Joyce^16^, which includes specimen NMB (Naturhistorisches Museum Basel, Switzerland) So.596, although the cervical vertebrae described herein are not mentioned in that paper.

### Description

The following cervical vertebrae are described considering the long axis of the centrum as horizontal, although this differs from the neutral position of these elements in the neck, which is defined as the position allowing the maximum overlap of the articular facets of the zygapophyses^4^.

**Figure 1:**
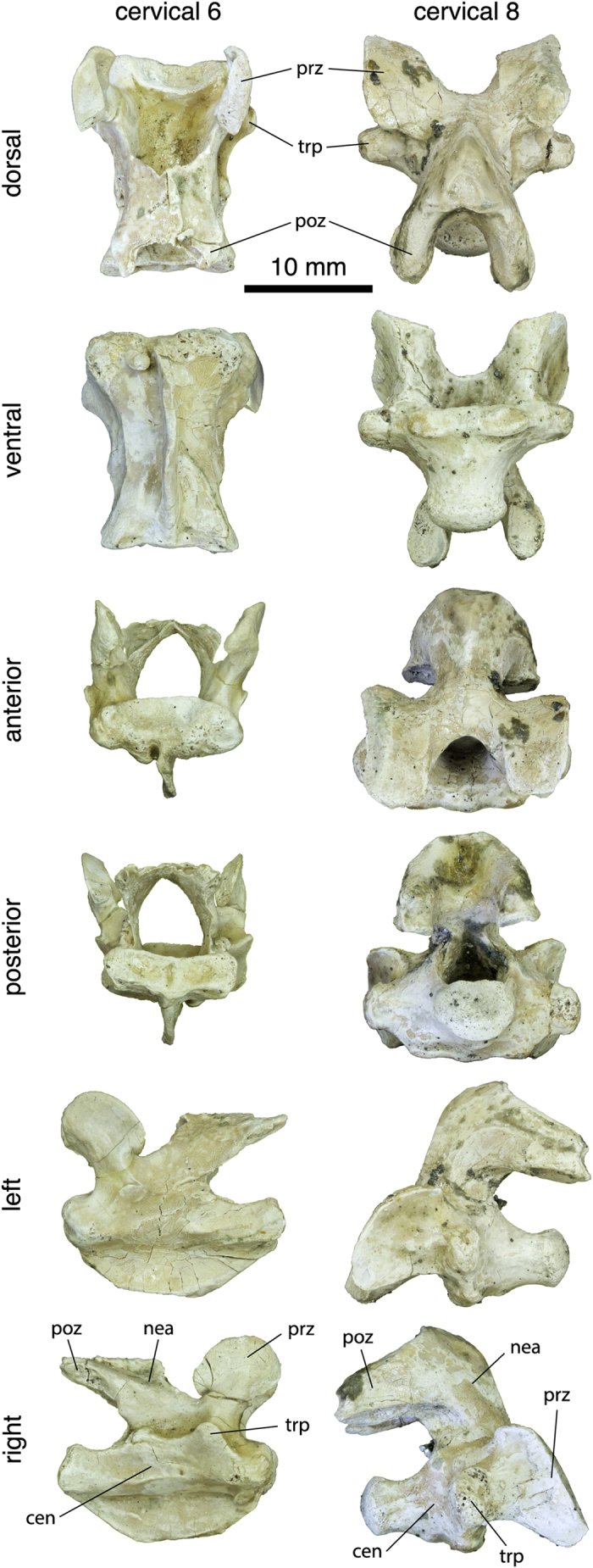
Cervical vertebrae of *Platychelys oberndorferi*. Sixth and eighth cervical vertebrae of specimen NMB So.596 in dorsal, ventral, anterior, posterior, left, and right views. Abbreviations: **cen**, centrum; **nea**, neural arch; **poz**, postzygapophysis; **prz**, prezygapophysis; **trp**, transverse process.

The centrum of the sixth cervical vertebra is longer than wide, flattened dorsoventrally, and rectangular in ventral view (Fig. 1). The ventral keel is well developed and most of its ventral margin is straight. The vertebra is biconcave, but the posterior articulation is relatively shallow. The single anterior articulation is much wider than high and faces anterodorsally. The areas ventral and ventrolateral to the anterior articulation are thickened and probably served for ligamentary and muscular attachments. The posterior articulation is double and much wider than high. It faces posteriorly. The two facets are moderately concave and are separated by a deep, narrow vertical furrow. The neural arch is high and deeply emarginated anterodorsally and posterolaterally. There is no neural spine. The pre- and postzygapophyses are widely spaced. The prezygapophyses are large rounded processes that project anterodorsally. The articular facets form a posterodorsal band on the medial surface of the prezygapophyses and face posteromedially and slightly dorsally. Anteroventral to this area, the medial surface of the prezygapophyses is slightly concave. The anterior margin of the prezygapophyses projects slightly medially. Compared to the prezygapophyses, the postzygapophyses are much more reduced and consist of short projections protruding from the posterodorsal margin of the neural arch. The articular facet is narrow and elongated, and faces ventrolaterally. The transverse process is only developed as a modest ridge projecting dorsolaterally and located ventral to the prezygapophysis. The transverse process is therefore located anteriorly along the centrum. Posterior to the transverse process, the ridge becomes less marked, but continues up to the posterolateral emargination of the neural arch. On the right side, this ridge thickens as it reaches the posterolateral emargination. This cervical vertebra has been erroneously reported as a seventh cervical by some authors^17^,^18^,^19^. This interpretation is however rejected herein since it is anatomically impossible to articulate this vertebra with the eighth cervical vertebra of the same individual. Several characteristics of this vertebra (including the great degree of dorsal flexion with the preceding vertebra and the strongly vertical orientation of the zygapophyses) support an identification as a sixth cervical (see Supplementary Data 1 for more detail).

The centrum of the eighth cervical vertebra is short and trapezoidal in ventral view (broader anteriorly; Fig. 1). There is no ventral keel, but a minute bulging occurs about halfway along the ventral surface of the centrum. The vertebra is biconvex. The anterior articulation is dorsoventrally flattened, much wider than high, and only developed as a modest lip on the anteroventral margin of the centrum. This articulation consists of double articular facets that face mostly ventrally. The two facets are separated by a low and broad sagittal ridge. The posterior articulation is a well formed, oval-shaped condyle being slightly wider than high and facing posteriorly. The neural arch is high and oriented strongly obliquely relative to the centrum, forming an angle of about 58° with the latter. There is no neural spine, but a minor sagittal ridge occurs posteriorly on the neural arch dorsal surface. A flat triangular area occurs on the dorsalmost part of the neural arch just dorsal to the stem of the postzygapophyses. This part of the vertebra was probably in contact with the ventral surface of the nuchal plate of the carapace, where a corresponding small, shallow depression is present. The zygapophyses are widely spaced and well developed. The prezygapophyses are relatively large, lobe-shaped (as seen in lateral view) structures projecting anterodorsally and extending much beyond the anterior end of the centrum. In dorsal aspect, they appear as longer than wide, flat processes. The articular surface on the dorsal surface of the prezygapophyses is shaped like a running track (an oval with straight sides) and is moderately convex. It faces mainly anterodorsally and only moderately medially. Posterior to the prezygapophyses, the neural arch is markedly waisted. The postzygapophyses consist of strong processes extending posteroventrally at 90° from the neural arch. A deep concavity facing posteriorly occurs between the base of the two postzygapophyses. A flat, oval-shaped articular surface covers the distal half of the ventral surface of each postzygapophysis. This articular surface faces ventroanteriorly and slightly laterally. The strong but short transverse process is located at the anterior end of the centrum, just dorsal to the level of the anterior central articulation. There is no evidence suggesting the presence of well-developed cervical ribs in this taxon.

### Comparison

In extant pleurodires, cervical vertebrae are usually narrow and tall with zygapophyses set close together or even fused in some species^2^,^3^. This morphology allows for maximal lateral flexion. In pleurodires, the transverse processes are well developed and form extensive insertion sites for the muscles of the longissimus group, which are responsible for the lateral flexion of the neck^1^. The transverse processes are usually located about midway along the vertebral centrum and often oriented somewhat posteriorly. The cryptodiran cervical vertebrae are more dorsoventrally compressed and somewhat wider, and the zygapophyses are usually widely spaced. This morphology is more adapted to sagittal flexion. In many cryptodires, movements in the sagittal plane are favored (and movements in the horizontal plane conversely restricted) by the presence of double central articular joints usually between cervicals 6 to 8, a condition called ginglymoidy^2^. The transverse processes are usually not well developed in cryptodires and are located anteriorly along the centrum. In these aspects, the rather low, wide cervical vertebrae with poorly developed, anteriorly positioned transverse processes, widely spaced zygapophyses, and ginglymoid articulations found in *Platychelys oberndorferi* are strikingly reminiscent of modern cryptodires.

Although similarities between the cervical vertebrae of *P. oberndorferi* and those of modern cryptodires are baffling, it is important to note that this resemblance is not total and that the cervical morphology in this species is remarkable in several aspects, notably in having a biconcave sixth cervical centrum, which is known to occur only as an individual variation in some emydids and one testudinid among modern cryptodires^2^. Patterns of cervical central articulation are actually quite variable among groups and even between species of cryptodires (notably in the position of the ginglymoid articulations and orientation of the articular surfaces). If our interpretation of the material described herein is correct, then the posterior cervical formula of *P. oberndorferi* would be 5))6{ {7{ {8) (see Williams^2^ for detail about the notation of the cervical formula). Therefore *P. oberndorferi* is clearly different from Americhelydia (chelonioids + chelydrids + kinosternoids), in which cervicals 5 to 8 (c5–c8) are usually procoelous. Like *P. oberndorferi*, testudinoids commonly have a biconvex eighth cervical, but these taxa usually have a procoelous sixth cervical and a biconcave seventh cervical. Finally, trionychians share with *P. oberndorferi* opisthocoelous articulations between the sixth and seventh and the seventh and eighth cervicals, but they lack the biconcave sixth cervical observed in *P. oberndorferi*.

Ginglymoidy is unknown in crown-group pleurodires, but it was proposed that *P. oberndorferi* may otherwise have a typical chelid cervical formula: (2((3((4((5))6))7((8), which would have suggested that this formula was plesiomorphic in pan-pleurodires^17^. However, since we reinterpreted the seventh cervical vertebra of previous authors as a sixth cervical, this proposition is no longer supported. The cervical formula of pelomedusoid (biconvex c2, procoelous c3–c8) is also different from that of *P. oberndorferi*. In pleurodires, the usually well-developed transverse process is located about midway along the centrum, which contrasts with the reduced, anteriorly positioned transverse process seen in *P. oberndorferi* and modern cryptodires.

Little is known about the morphology of cervical vertebrae in other platychelyids. A single, incomplete eighth cervical vertebra is associated with the holotype of *Notoemys oxfordiensis* (Fuente and Iturralde-Vinent, 2001)^19^. The anterior part of this vertebra is lacking, but the posterior central articulation and the postzygapophyses are congruent with the eighth cervical of *P. oberndorferi*: neural arch strongly oblique relative to centrum; strongly developed and widely spaced postzygapophyses extending at 90° from the neural arch; and convex posterior central articulation. Cervical vertebrae 1 to 4 are known in *Notoemys laticentralis* Cattoi and Freiberg, 1961^19^,^20^,^21^,^22^. The atlas is biconcave, whereas the c2–c4 are opisthocoelous. Cervical vertebrae 2 to 4 are relatively low elements with anteriorly placed transverse processes (proportionally more developed than on the sixth cervical of *P. oberndorferi*) and widely spaced zygapophyses. This morphology is congruent with that of the sixth cervical vertebra of *P. oberndorferi*. Therefore, we can expect the cervical morphology (and possibly the neck retraction) exhibited by *Notoemys laticentralis* and *Platychelys oberndorferi* to be general for platychelyids.

### Phylogenetic perspective

*Proterochersis robusta* from the Late Triassic of Germany was long considered the oldest and most basal pan-pleurodiran turtle, but this taxon was recently reinterpreted as a basal stem turtle^7^,^23^,^24^,^25^. Platychelyids are, however, universally retrieved as stem pleurodires and are therefore the oldest pan-pleurodires^16^. In this context, the fact that the neck of *Platychelys oberndorferi* was adapted toward flexion in the vertical plane like modern cryptodires is somewhat counter-intuitive. However, many characteristics of platychelyid turtles agree with their placement as stem pleurodires (sutural articulation of pelvis with shell, loss of medial contact of mesoplastra, well-developed anal notch, single gular scale^16^).

Most stem turtles as well as numerous early stem cryptodires (e.g., xinjianchelyids, plesiochelyids) lack formed cervical articulations. Formed cervical centra actually appeared independently several times within Testudinata during the Cretaceous, notably in some stem turtles (*Mongolochelys efremovi* and meiolaniformes), in some derived baenids, and in sinemydids and macrobaenids along the stem of crown-group cryptodires^7^,^26^. Since Gaffney *et al*.^27^, *P. oberndorferi* has been repeatedly scored as having formed cervical articulations without impact on its phylogenetic position. As a matter of fact, with *Proterochersis robusta* no longer being considered a stem pleurodire, formed cervical articulations were considered to be primitive for pan-pleurodires as a whole^7^,^23^. As early as the Late Jurassic, platychelyids are therefore the first turtles to develop formed cervical articulations.

Until Lapparent de Broin and Murelaga^17^ first mentioned its presence in *P. oberndorferi*, ginglymoidy was thought to be exclusive to cryptodires^2^. Early occurrences of cryptodiran ginglymoidy were reported in Late Cretaceous pan-trionychians (adocids and nanhsiungchelyids^28^,^29^) and testudinoid lindholmemydids^30^. Ginglymoidy is found in most modern cryptodires, although variations occur^2^. For example, ginglymoidal central articulations are sometimes absent as an intraspecific variation in chelydrids and weakly developed or often absent in chelonioids^2^. In other cryptodires, the position and number of ginglymoidal central articulations are relatively variable intra- and interspecifically. The same can be said of the orientation of articular surfaces and of the presence and position of biconcave and biconvex cervical vertebrae^2^.

Ginglymoidy was first scored in a global phylogenetic analysis of turtles by Sterli and Fuente^31^. This matrix is based on that of Joyce^7^, as modified by Sterli^32^ and Sterli and Fuente^26^, and was subsequently refined through addition of new characters and modification of some scorings^9^,^33^,^34^,^35^. We have used the last version of this matrix^35^ updated for cervical characters in order to test the impact of the peculiar cervical morphology of *P. oberndorferi* on the phylogenetic position of this taxon within Testudines. Our analysis (see Methods) resulted in 1,128 trees of 860 steps (CI = 0.348; RI = 0.797). The strict consensus of these 1,128 trees is strictly identical to the one resulting from the analysis of the original matrix^35^. Therefore, scoring the presence of a biconcave cervical vertebra (Cervical vertebra G), the presence of ginglymoidal joints between the sixth and seventh and between the seventh and eighth cervicals (Cervical articulation J, Cervical articulation L), and the cryptodire-like postzygapophyses of the eighth cervical vertebra (Cervical vertebra I) in *P. oberndorferi* has no effect on the phylogenetic position of either this species, platychelyids, or pan-pleurodires. This strongly supports the idea that the cervical morphology of *P. oberndorferi* is convergent with that of crown-group cryptodires.

### Biomechanical analysis

In modern cryptodires, the base of the neck bends strongly downward between the first thoracic vertebra and the eighth cervical vertebra. This morphology results in a strong ventral bending of the postzygapophyses of the eighth cervical vertebra that is characteristic of cryptodires^12^ and found in *Platychelys oberndorferi* as well. The anterior central articulation of the first thoracic vertebra is already oriented ventrally in many cryptodires, which means that the initial reorientation of the neural passage at the base of the neck occurs between the first thoracic and the eighth cervical vertebrae. In *P. oberndorferi*, the anterior articulation of the first thoracic vertebra is oriented anteriorly and the neural canal is reoriented downward within the eighth cervical (60° angle). The articulation of the eighth cervical of specimen NMB So.596 with the first thoracic vertebra of the associated shell clearly shows the downward orientation of the base of the neck in *P. oberndorferi*. In cryptodires in which the base of the neck bends downward, the neck usually levels out around the fifth and sixth vertebrae. The reorientation of the neural passage can occur within (bent neural canal) and/or between (central articulations oblique relative to centrum) the vertebrae. The emargination between pre- and/or postzygapophyses is usually deeper in the second case. In the sixth cervical vertebrae of *P. oberndorferi*, the anterior part of the neural canal is bent dorsally, the anterior articulation is oriented anterodorsally, and the neural arch is deeply emarginated between the prezygapophyses. From the above, it follows that the neutral position of the posterior part of the neck in *P. oberndorferi* is similar to the one observed in cryptodires: the base of the neck bends markedly downward before levelling out anteriorly.

The articular contacts between the postzygapophyses of the eighth cervical and prezygapophyses of the first thoracic vertebrae are sub-horizontal and moderately elongated. Movements in the vertical plane are favored, but they are relatively limited in extent. Minor horizontal movements were also probably possible. In contrast, the prezygapophyseal articular surfaces of the eighth cervical vertebra are convex and much more developed. The articular surfaces are greatly elongated and probably allowed for a great angular movement in the vertical plane. Additionally, movements between the seventh and eighth cervical vertebrae were limited to the vertical plane by the ginglymoid central articulation. The central articulation between the sixth and seventh cervical vertebrae is also ginglymoid, similarly limiting movement to the vertical plane. The postzygapophyseal articulations of the sixth cervical are reduced and oriented mostly laterally, which is congruent with a limitation of the movements to the vertical plane. Given the small surface of the postzygapophyseal articulations on the sixth cervical, the degree of movement between the sixth and seventh cervical vertebrae was probably reduced. The anterior central articulation of the sixth cervical vertebra is a dorsoventrally flattened, deep oval cavity. This articular surface is oriented anterodorsally relative to the long axis of the centrum, forming an angle of about 35°. This indicates a major reorientation of the neural passage between the fifth and sixth cervical vertebrae. This is also apparent in the deep emargination of the neural arch between the prezygapophyses of the sixth cervical. The prezygapophyseal articular surface of the sixth cervical is elongated and moderately convex. This suggests a relatively important degree of movement, although the latter was restricted to the vertical plane as indicated by the vertical orientation of the prezygapophyses.

**Figure 2:**
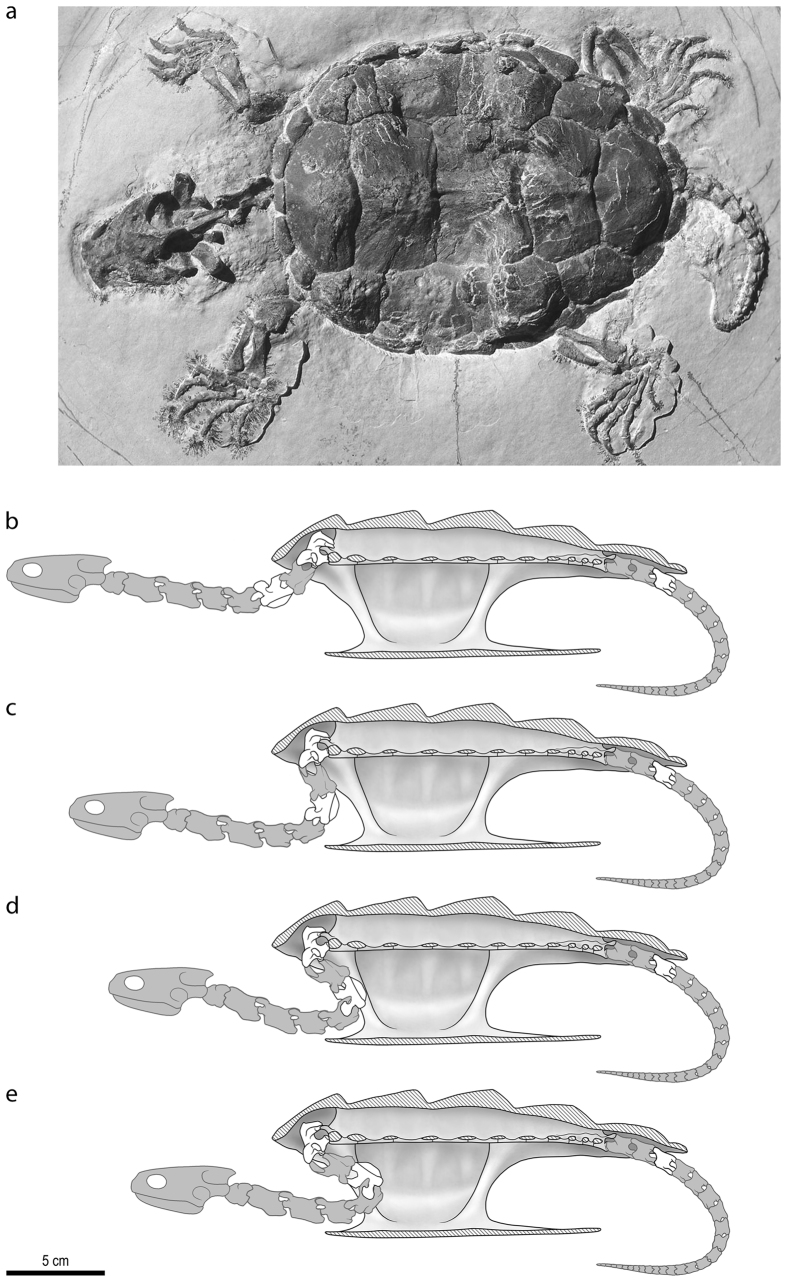
Neck mobility in *Platychelys oberndorferi*. (**a**) Only-known complete individual of *Platychelys oberndorferi* from the Late Jurassic of Eichstätt, Germany (undescribed; coll. Stefan Schäfer, Puchheim, Germany), illustrating the peculiar morphology of this species [photo by H. Tischlinger]. The biomechanichal reconstructions (**b**–**e**) represent specimen NMB So.596, for which the shell, two cervical vertebrae, and one caudal vertebra are known (elements in white). Other parts of the skeleton (elements in grey) where either derived from other specimens or reconstructed based on adjoining elements (see Methods). (**b**) Maximal protraction of the neck. (**c**) Neutral position of the neck (maximum overlap of zygapophyses). (**d**) Probable maximal retraction of the neck. (**e**) Extreme hypothesis of maximal retraction of the neck (see Biomechanical analysis). Reconstructions by P. Röschli.

With only the sixth and eighth cervical vertebrae preserved, it is somewhat difficult to reconstruct the neck mobility of *P. oberndorferi* with complete confidence. However, these elements clearly indicate a trend toward the restriction of movement to the vertical plane in the posterior part of the neck and the possibility for the neck to be withdrawn in the vertical plane. Biomechanical reconstructions (Fig. 2) were made in order to further investigate neck movements in *P. oberndorferi* (see Methods). The greatly developed convex prezygapophyseal articular surfaces of the sixth cervical indicate a high degree of angular movement between the fifth and sixth cervical vertebrae, corresponding to a first kink in the cervical series. A second kink is formed by the strong ventral reorientation of the neural canal within the eighth cervical (ventrally oriented anterior central articulation) and the extensive development of the prezygapophyses of the eighth cervical suggesting an important degree of movement between the seventh and eighth cervical vertebrae. Based on the shape of the vertebrae, the degree of movement along articular joints, the available space between the carapace and plastron, and the comparison with modern turtles, multiple retraction scenarios were tested (Fig. 2). Some modern turtles are able to dislocate some cervical joints during retraction^4^, but it is unknown whether *P. oberndorferi* was able to do so. A conservative approach is therefore to maintain contact between articular surfaces during retraction (Fig. 2d). A more extreme scenario was also tested. Some cervical joints at the base of the neck were dislocated and the dorsal flexion of the c5–c6 joint was pushed up to the point where there is no more contact between the zygapophyses (Fig. 2e). The difference between each scenario is actually quite small, and in both cases the head and anterior part of the neck cannot be completely withdrawn within the shell. This suggests that the complex cervical morphology allowing vertical neck retraction in *P. oberndorferi* evolved for other reasons than protection.

## Discussion

*Platychelys oberndorferi* is a peculiar form among Late Jurassic turtles. Its carapace is characterized by the presence of three longitudinal rows of high knobs located at the growth center of vertebral and pleural scales. The striking resemblance with the matamata (*Chelus fimbriatus*) and alligator snapping turtle (*Macrochelys temminckii*) was noted by many workers^5^,^10^,^15^,^16^,^36^. The resemblance is actually not limited to the shell, as notably illustrated by a beautifully preserved, privately held fossil from Eichstätt^37^,^38^ (Fig. 2a). Like the two aforementioned extant species, *Platychelys oberndorferi* is also characterized by the presence of a wide costovertebral tunnel (a feature of uncertain function that is only known in platychelyids, the matamata, and chelydrids), large hyoids, a relatively wide skull with orbits placed close to the snout tip, and powerful limbs with strong claws. This strongly suggests that *P. oberndorferi* had a life style very similar to that of the extant matamata and alligator snapping turtle^16^. These turtles are ambush predators living on the bottom of shallow waters where their irregular carapace and skin appendages help them camouflage among vegetation^39^.

Most modern aquatic turtles capture prey or food item by rapidly extending the neck and projecting the head forward while the rest of the body usually remains stationary. The forward thrust of the head is particularly fast in some species, notably *Chelus fimbriatus* and *Chelydra serpentina*^40^. The opening of the jaws and fast expansion of the oropharyngeal volume during food uptake generate a suction force that compensates the bow wave created by the approaching head^41^. In the common snapping turtle (*Chelydra serpentina*), the suction force only compensates for the bow wave and the position of the prey is not affected by the fast approaching head and opening mouth^42^. Therefore, this species is more accurately described as a fast ram feeder. The feeding kinematics of the alligator snapping turtle (*Macrochelys temminckii*) has not yet been studied in detail, but observations suggest that the feeding pattern in this species is quite comparable to that of *Chelydra serpentina*^39^. In most other aquatic turtles, it is assumed that the suction force not only compensates for the approaching head, but also attracts the food toward to oral cavity at least to some extent^40^,^41^,^43^. In contrast, the matamata (*Chelus fimbriatus*), as well as other chelids and some trionychids, are able to generate an enormous suction force thanks to a highly derived cranio-mandibular morphology, an extremely developed hyoid apparatus and musculature, and a greatly distensible oesophagus^44^,^45^. The matamata is therefore best described as a very specialized suction feeder.

**Figure 3:**
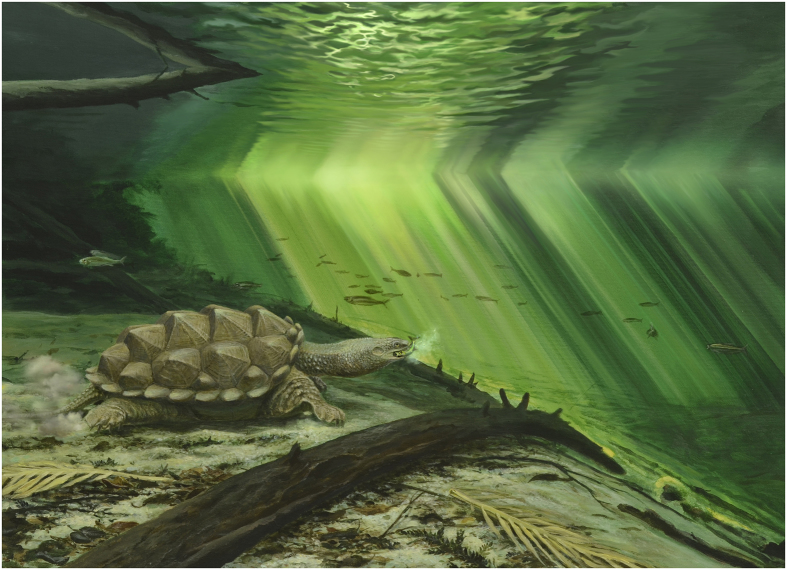
Life reconstruction of *Platychelys oberndorferi* in its palaeoenvironment. This reconstruction represents *Platychelys oberndorferi* as a fast ram feeder and emphasizes the resemblance of this taxon with the extant matamata (*Chelus fimbriatus*) and snapping turtles (*Chelydra serpentina* and *Macrochelys temminckii*). Artwork by P. Röschli.

The cranial and cervical anatomy of the matamata and snapping turtles is markedly different, but their external morphology and feeding behavior are remarkably convergent. These turtles are ambush predators that thrust their head forward with high velocity to capture prey. Fast forward projection of the head necessitates accurate control of the multi-jointed head-cervical system^1^. In some long-necked suction-feeding chelids, the head is apparently pulled forward by the great suction force generated by the gaping jaws, simplifying the necessary control of the head-cervical system^46^. In other turtles relying less on suction to capture prey, such as chelydrids, other control mechanisms must be in place^1^. We suggest that, by strongly reducing degrees of movement at the base of the neck, partial vertical neck retraction in *P. oberndorferi* evolved primarily to enable and control fast forward projection of the head, hence improving capture of darting prey (Fig. 3).

It is usually assumed that turtles developed neck retraction for protective reasons. However, the functional origin of neck retraction mechanisms in turtles has never been properly investigated. It is now widely accepted that turtles originated from terrestrial, possibly fossorial, ancestors during the Permian^47^,^48^ and that the vast majority of stem turtles were similarly terrestrial^49^,^50^. These stem turtles were not able to retract their neck like modern forms, but they could apparently tuck their head laterally possibly in order to gain some protection^4^. By the Middle Jurassic, several stem turtles became adapted to aquatic environments^51^,^52^, and there is a consensus to consider that crown-group turtles (uniting the modern-day pleurodires and cryptodires) are ancestrally aquatic. Modern neck retraction mechanisms subsequently evolved independently in pleurodires and cryptodires^4^,^12^. And it is noteworthy that neck retraction appeared in the aquatic medium in both groups (modern tortoises inherited it from their aquatic ancestors).

The horizontal folding of pleurodires actually offers little more protection for the head and neck than the lateral head tuck of stem turtles, raising questions on the advantage of developing such a complex cervical system. It is possible that the pleurodiran mode of neck retraction evolved as a way to accommodate increasingly longer necks and that a condition resembling that of pleurodires (only less derived) was ancestral to all crown group turtles^12^. In cryptodires, the shift from the ancestral condition (whether lateral head tucking or pleurodiran-like) to the retraction in the vertical plane involved significant morphological changes that resulted in the loss of the ability to withdraw the neck laterally. However, in the case of cryptodires, the protection of the head is effective only when the head is completely withdrawn within the shell and vertical retraction fully achieved. Partial vertical neck retraction, such as the one observed in *P. oberndorferi*, offers no additional value for protection. Therefore, it is unlikely that protection was a driving force in the evolution of the complex double-bend vertical neck retraction mechanism typical of modern cryptodires.

*Platychelys oberndorferi* suggests that a mechanism of neck retraction very similar to what is known in modern cryptodires can evolve for reasons that have apparently nothing to do with protection, but rather with improving prey capture abilities in the aquatic medium. This case offers a likely explanation to the origin of complex vertical neck retraction mechanism of cryptodires. Neck retraction could have evolved gradually as a way to enable fast forward thrust of the head during prey capture under water. Partial neck retraction would already provide an advantage, as demonstrated by the very specialized ecological niche occupied by *P. oberndorferi*. Once a primary mechanism of vertical neck retraction was in place, it would be easier to obtain complete head withdrawal for protection via natural selection.

We therefore suggest that vertical neck retraction may have evolved in cryptodires primarily in order to enable fast forward projection of the head during underwater feeding, and that protection of the head by complete withdrawal within the shell in the vertical plane is actually an exaptation^53^. This hypothesis should of course be tested in future studies, notably by exploring the mechanisms that control fast forward projection of the head during feeding in modern forms. This is however the first time that a plausible functional origin is proposed for the cryptodiran mode of neck retraction. We hope that this study will inspire other to continue exploring the evolution of cervical vertebrae in early crown group turtles.

## Methods

### Material

NMB So.596 (formerly sor.67.9) consists of a relatively complete shell and articulated pelvic girdle from the Late Jurassic of Solothurn (Canton of Solothurn, Switzerland). The specimen is housed at the Naturhistorisches Museum in Basel (Switzerland) at least since 1862, as it is first mentioned in the annual report of this institution for that year. This specimen was studied and illustrated by several authors^54^,^55^,^56^. Acid preparation at the American Museum of Natural History revealed the presence of three associated vertebrae (see Supplementary Data 1). Two can be confidently identified as the sixth and eighth cervical vertebrae and are described herein for the first time, although they have been elusively mentioned in the literature at several occasions^17^,^18^,^19^,^56^,^57^ (see Supplementary Data 1). Since they have never been properly described, the known caudal vertebrae of *Platychelys oberndorferi* are briefly discussed and figured in the attached supplementary information (Supplementary Data 1).

### Geological setting

Specimen NMB So.596 comes from one of the former quarries that were exploited in the vicinity of Solothurn (Canton of Solothurn, Switzerland), one of the richest known locality in Europe for Late Jurassic turtles^5^,^36^,^54^,^58^,^59^,^60^. The exact locality and stratigraphic horizon in which NMB So.596 was found are uncertain. The only indications on the old label accompanying the specimen are “Solothurn” and “Ptérocérien”. The latter would correspond to the lower part of the Kimmeridgian^61^. However, the turtles from Solothurn are usually considered to come from the Solothurn Turtle Limestone, which corresponds to the uppermost member of the Reuchenette Formation (Autissiodorensis ammonite zone, late Kimmeridgian)^58^.

### Phylogenetic analysis

In order to test the potential impact of the new information presented herein on the phylogenetic position of *Platychelys oberndorferi* within Testudines, we have updated the last available global matrix of discrete characters for fossil and extant turtles^35^ (Supplementary Data 2). Scorings for *P. oberndorferi* were changed as follows (previous scorings in parentheses): Cervical vertebra G: 1 (?); Cervical articulation I: 0 (–); Cervical articulation J: 1 (–); Cervical articulation K: 0 (–); Cervical articulation L: 1 (–); Cervical vertebra I: 1 (?); Cervical vertebra K: 0 (?); Caudal B: 1 (?). The analysis was run with TNT v.1.5-beta^62^ using the same settings as in the original analysis, including the molecular backbone constraining the relationships of cryptodires^35^. A heuristic search using tree-bisection-reconnection (TBR) swapping algorithm with 1000 random addition replicates and 10 trees saving per replicates followed by a second round of TBR swapping resulted in 1,128 trees of 860 steps (CI = 0.348; RI = 0.797).

### Biomechanical reconstruction

The reconstructions provided in Fig. 2 are based on our analysis of the morphology of the cervical vertebrae in *Platychelys oberndorferi* and our interpretation of the degree of movement at their joints. A detailed comparison with modern cryptodires, notably *Macrochelys temminckii*, was carried out as part of this work. The shell morphology and notably the available space between the carapace and plastron have been directly reconstructed from the marginally deformed shell associated with the cervical vertebrae described herein (NMB So.596), ensuring that the proportions are as close as possible to reality. The morphology of the anterior part of the neck (unknown in *P. oberndorferi*) has been derived from that of the closely related platychelyid *Notoemys laticentralis*^22^. Head shape and proportion are based on an undescribed complete specimen of *Platychelys oberndorferi* held in private hand^37^,^38^. Similar comparisons were made to produce the life reconstruction given in Fig. 3.

### Data availability

The updated data matrix is available as supplementary information (Supplementary Data 2).

## Additional Information

**How to cite this article**: Anquetin, J. *et al*. A Jurassic stem pleurodire sheds light on the functional origin of neck retraction in turtles. *Sci. Rep.* **7**, 42376; doi: 10.1038/srep42376 (2017).

**Publisher's note:** Springer Nature remains neutral with regard to jurisdictional claims in published maps and institutional affiliations.

## Supplementary Material

Supplementary Data 1

Supplementary data 2
