# Supplementary material for: A Jurassic stem pleurodire sheds light on the functional origin of neck retraction in turtles
Source: Sci Rep. 2017 Feb 16;7:42376. doi: 10.1038/srep42376 (PMC5312562; doi:10.1038/srep42376)
Supplement: Supplementary Data 1 [file srep42376-s1.pdf]

SUPPLEMENTARY DATA 1

A Jurassic stem pleurodire sheds light on the functional origin of neck retraction in turtles

Jérémy Anquetin[1,2], Haiyan Tong[3], Julien Claude[4]

[1]JURASSICA Museum, Porrentruy, Switzerland

[2]Department of Geosciences, University of Fribourg, Fribourg, Switzerland

[3]Palaeontological Research and Education Centre, Mahasarakham University, Mahasarakham, Thailand

[4]Institut des Sciences de l'Evolution de Montpellier, UMR 5554 CNRS/UM/IRD/EPHE, Montpellier, France

In the present document, we provide additional background information regarding the cervical and caudal vertebrae of the Late Jurassic turtle *Platychelys oberndorferi*.

Previous work

Vertebral elements of *Platychelys oberndorferi* have been previously discussed by several authors. Bräm[1] first mentioned two vertebrae that were found during the acid preparation of specimen NMS 8686–8690 (formerly NMS 56). These were initially identified as cervicals, but were only briefly discussed[1]. However, Lapparent de Broin and Murelaga[2] and Gaffney et al.[3] correctly reattributed these to caudals.

Other authors specifically mentioned cervical vertebrae for *Platychelys oberndorferi*. Meylan[4] first stated that cervical articulations were formed in specimen NMS 8691 (formerly NMS 57), but this is incorrect since no vertebrae are associated with this specimen. Referring to the material mentioned by Meylan[4], Lapparent de Broin and Murelaga[2] stated that the seventh cervical vertebra was biconcave and the eighth biconvex, and that the articulation between these two vertebrae was formed by a double condyle. These observations were re-stated by Lapparent de Broin[5], who also added that the zygapophyses were well separated and that the transverse process was located anteriorly along the vertebral centrum in this taxon. Fuente and Iturralde-Vinent[6] mentioned a biconvex eighth cervical vertebra in an uncatalogued specimen of *Platychelys oberndorferi* from the NMB.

Gaffney et al.[3] accepted some of the conclusions from previous authors, notably Lapparent de Broin and Murelaga[2]. They scored *Platychelys oberndorferi* as having the 'chelid condition' for cervical articulations, which is notably characterized by a biconcave seventh and a biconvex eighth cervical vertebrae. They also scored postzygapophyses as being separated, but only mentioned one known cervical vertebra for this taxon. However, Gaffney et al.[3] did not mention double cervical articulations for *Platychelys oberndorferi*. To the exception of Meylan[4], none of the aforementioned authors clearly identified the specimen(s) they were drawing conclusions from.

This confusing situation led Cadena and Joyce[7] to erroneously conclude that the two cervical vertebrae discussed by Lapparent de Broin[5] and Gaffney et al.[3], one biconcave and the other biconvex, were actually the two (caudal) vertebrae initially described by Bräm[1]. Our interpretation is that the cervical vertebrae mentioned by Meylan[4], Lapparent de Broin and Murelaga[2], Lapparent de Broin[5], Fuente and Iturralde-Vinent[6], and Gaffney et al.[3] are actually those found associated with specimen NMB So.596, which are fully described in the main text for the first time. This interpretation was recently confirmed to the authors by P. Meylan (pers.comm., 2016). Specimen NMB So.596 was borrowed by E. S. Gaffney and prepared at the AMNH by P. A. Meylan during the 1980s. Three vertebrae (two cervicals and one caudal) were found during preparation of this specimen and used notably by Gaffney et al.[3] for character coding (E. S. Gaffney, pers. comm., 2015). The shell of specimen NMB So.596 was returned to Basel several years ago, but the associated vertebrae were only returned in 2015. Several specialists probably saw this material when it was still in New York. For example, F. de Lapparent de Broin (pers. comm., 2015) confirmed that the conclusions of Lapparent de Broin and Murelaga[2] and Lapparent de Broin[5] on *Platychelys oberndorferi* were based on photographs of the cervical vertebrae of specimen NMB So.596, which were taken by M. S. de la Fuente in 1996 at the AMNH.

Description of caudal vertebrae

For the sake of completeness, the known caudal vertebrae of *Platychelys oberndorferi* are briefly described and illustrated here. Bräm[1] illustrated and briefly discussed two vertebrae found associated with specimen NMS 8686–8690 (formerly NMS 56). These two vertebrae (numbered NMS 8687 and NMS 8688) differ from one another in morphology (Supplementary Figure 1). A third caudal vertebra was found associated with specimen NMB So.596 (see above) and is morphologically more similar to NMS 8688.

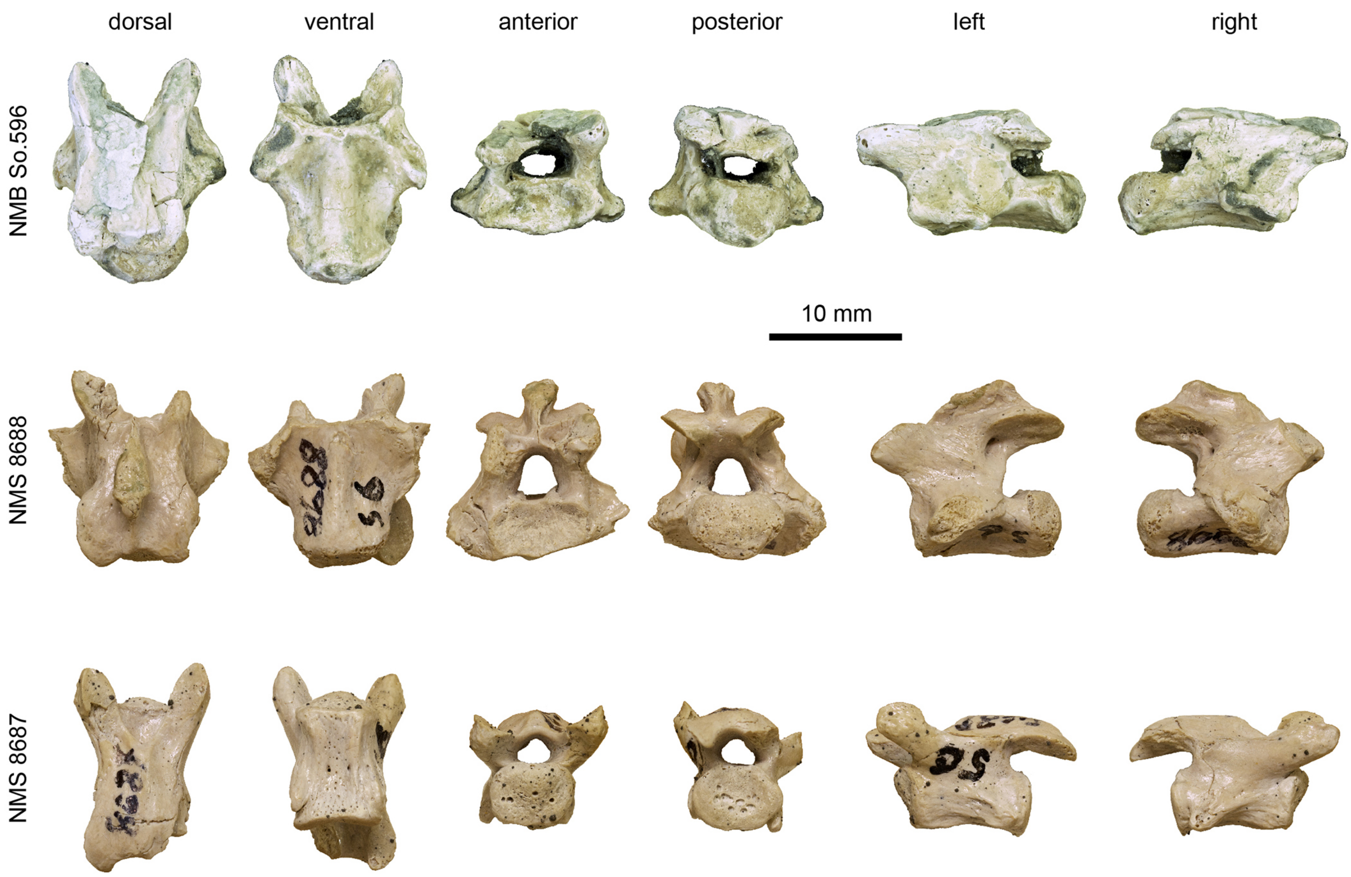

Supplementary Figure 1 I Caudal vertebrae of *Platychelys oberndorferi*. Caudal vertebrae NMB So.596, NMS 8687, and NMS 8688 in dorsal, ventral, anterior, posterior, left, and right views.

NMS 8688 consists of a relatively high vertebra with a well-developed neural arch. The dorsal part of NMB So.596 is damaged, which makes the vertebra appearing less high than NMS 8688, but otherwise the morphology is very similar. The centrum is somewhat longer than wide (less so in NMS 8688) and flattened dorsoventrally. There is no ventral keel, but a broad, low ridge is present. NMS 8688 and NMB So.596 are procoelous. The anterior central articulation is significantly wider than high and faces anteriorly. In NMB So.596, it is even slightly wider than the posterior width of the centrum. The posterior central articulation is clearly formed, less dorsoventrally flattened than the anterior one, and vaguely trilobed in posterior view. It faces posteriorly. There are no posteroventral articular facets for chevrons, so the latter were probably absent. The transverse process is strongly developed and located anteriorly along the centrum. It forms a thick alar projection extending posterolaterally from the lateral margin of the anterior central articulation. The transverse process terminates bluntly in NMB So.596, but in NMS 8688 the lateral part of each

transverse process is broken, which indicates an even greater development. The neural arch is well developed dorsally and is fused to the centrum. A moderately high, but thick neural spine is present. In NMS 8688, the neural spine is only developed along half the length of the vertebra, and its dorsal margin is thickened and rough. The neural spine is partly damaged in NMB So.596, but it apparently extends on most of the length between the two zygapophyseal emarginations, and its dorsal part widens and forms a tabular surface, possibly along most of this length. The zygapophyses are widely spaced. The prezygapophyses face dorsomedially, whereas the postzygapophyses face ventrolaterally and do not project posteriorly past the level of the posterior central articulation. A pair of large foramina is present anteroventral to the postzygapophyses in NMS 8688. The existence of these foramina in NMB So.596 is impossible to assess because the concerned area is masked by the broken postzygapophyses. To our knowledge, such foramina are not generally present in turtle caudal vertebrae and their nature in *Platychelys oberndorferi* remains unknown.

The morphology of these two vertebrae (high neural arch, short centrum, neural spine present, and large transverse process) suggests that they are proximal caudals. Based on the above description (shorter centrum, larger transverse process), NMS 8688 is probably more proximal than NMB So.596. The postzygapophyses of the last sacral vertebra of NMB So.596 are sub-vertical and face mostly laterally, which contrasts with the dorsomedially facing prezygapophyses of the two caudals NMS 8688 and NMB So.596. Therefore, none of these two proximal caudals is the first caudal vertebra.

NMS 8687 consists of a low, moderately elongated vertebra. The centrum is longer than wide and sub-cylindrical in cross-section. There is no keel nor midline ridge on the ventral surface of the centrum, but a pair of small foramina is present at mid-length. Bräm[1] described this vertebra as opisthocoelous, but the central articular surfaces are actually not fully formed. The dorsomedial part of the anterior central articulation is convex, but the rest of the articular surface is flat. Similarly, the dorsomedial part of the posterior central articulation is moderately concave, whereas the rest of the articular surface is flat. The poorly developed transverse process consists of a lateromedially flattened projection extending posteroventrally and flanking the posterior half of the centrum. The neural arch is relatively low and fused to the centrum. There is no neural spine, but a low ridge is present on the dorsal surface of the neural arch. The zygapophyses are well developed, widely spaced, and extend anteriorly and posteriorly beyond the level of the central articulations. The prezygapophyses face dorsomedially, whereas the postzygapophyses face

ventrolaterally. The morphology of NMS 8687 (elongate vertebrae, low neural arch, posteriorly located transverse process) indicates that this vertebra was located more distally than NMS 8688 and NMB So.596 along the caudal series.

Identification and orientation of the cervical vertebrae

Of the two cervical vertebrae described in this study, one can unmistakably be recognized as an eighth cervical vertebra. Similarly shaped eighth cervicals where the postzygapophyses bend strongly ventrally relative to the plan of the centrum or neural arch are relatively common in cryptodires[8]. This results in the downward orientation of the vertebral column at the base of the neck. In the case at hand, this eighth cervical vertebra articulates fairly well with the first thoracic vertebra of the associated shell (NMB So.596), supporting this interpretation.

The second cervical vertebrae of specimen NMB So.596 has been previously reported as a seventh cervical[2,5,6]. However, this vertebra does not articulate well with the associated eighth cervical vertebra. This is not simply a problem of postmortem deformation. The broad, low sagittal ridge separating the two articular facets of the anterior central articulation of the eighth cervical cannot fit in the posterior central articulation of the other cervical vertebra. More strikingly, the reduced, narrow articular facet of the postzygapophyses of the second cervical vertebra clearly does not match the very large articular facet of the prezygapophyses of the eighth cervical vertebra. The interpretation of the second vertebra as a seventh cervical is therefore refuted herein. Several characteristics of this second vertebra suggest that it is relatively distally placed in the cervical series: ginglymoid posterior articulation; well-developed ventral keel; strong dorsal bending of the neural passage anteriorly; and zygapophyses constraining the movement in the vertical plane. Based on these features and comparisons with morphologically similar modern cryptodires (especially chelydrids and testudinids), we interpret this vertebra as a sixth cervical. The large and nearly vertical prezygapophyses of this vertebra provide further support for its identification as a sixth cervical. In many cryptodires, the prezygapophyses the sixth cervical are more developed and vertically oriented than those of the seventh or eighth cervical because the neck is kinked at this level.

Orienting the eighth cervical vertebra is relatively easy since this element can be articulated with the first thoracic vertebra of the associated shell. The shell of *Platychelys oberndorferi* is relatively low and the series of thoracic vertebrae is horizontal. The anterior central articulation of the first thoracic vertebra faces anteriorly. When the eighth cervical and

first thoracic vertebrae are articulated in a neutral position (vertebral position with the greatest zygapophyseal overlap[9]), the long axis of the centrum of the eighth cervical vertebra dips slightly anteriorly. Passing through the eighth cervical, the neural canal bends at a 60° angle and continues horizontally toward the rear.

The orientation of the sixth cervical vertebra is more difficult to interpret. In many cryptodires, the base of the neck is oriented ventrally and the neck levels out in the area of the fifth and sixth cervical vertebrae. Given how the neural canal bends in the eighth cervical, a similar condition is probably also present in *Platychelys oberndorferi*. The neural canal runs relatively straight within most of the sixth cervical vertebra, but anteriorly it bends at a 35° angle dorsally, based on the orientation of the anterior central articulation. This corresponds fairly well to the levelling out observed between the fifth and sixth cervical vertebrae of the extant alligator snapping turtle (*Macrochelys temminckii*). This kink in the cervical series is also apparent in the deep anterodorsal emargination of the neural arch of the sixth cervical vertebra, which suggests an important angle between the fifth and sixth cervicals. Ultimately, it is impossible to determine the correct orientation of the sixth cervical vertebra without the seventh cervical. However, our biomechanical interpretation (see main text) suggests that the long axis of the sixth cervical vertebra was probably more or less vertical in a neutral position.

Institutional abbreviations. AMNH, American Museum of Natural History, New York, USA; NMB, Naturhistorisches Museum, Basel, Switzerland; NMS, Naturmuseum, Solothurn, Switzerland.

References

1. Bräm, H. Die Schildkröten aus dem oberen Jura (Malm) der Gegend von Solothurn. *Schweiz. Paläontol. Abh.* **83,** 1–190 (1965).
2. Lapparent de Broin, F. de & Murelaga, X. Turtles from the Upper Cretaceous of Laño (Iberian Peninsula). *Estud. Mus. Cienc. Nat. Alava* **14,** 135–211 (1999).
3. Gaffney, E. S., Tong, H. & Meylan, P. A. Evolution of the side-necked turtles: the families Bothremydidae, Euraxemydidae, and Araripemydidae. *Bull. Am. Mus. Nat. Hist.* **300,** 1–698 (2006).
4. Meylan, P. A. Skeletal morphology and relationships of the Early Cretaceous side-necked turtle, *Araripemys barretoi* (Testudines: Pelomedusoides: Araripemydidae), from the Santana Formation of Brazil. *J. Vertebr. Paleontol.* **16,** 20–33 (1996).

5. Lapparent de Broin, F. de. The oldest pre-Podocnemidid turtle (Chelonii, Pleurodira), from the early Cretaceous, Ceará state, Brasil, and its environment. *Treb. Mus. Geol. Barc.* **9,** 43–95 (2000).
6. Fuente, M. S. de la & Iturralde-Vinent, M. A new pleurodiran turtle from the Jagua Formation (Oxfordian) of western Cuba. *J. Paleontol.* **75,** 860–869 (2001).
7. Cadena, E. A. & Joyce, W. G. A review of the fossil record of turtles of the clades Platychelidae and Dortokidae. *Bull. Peabody Mus. Nat. Hist.* **56,** 3–20 (2015).
8. Werneburg, I., Wilson, L. A. B., Parr, W. C. H. & Joyce, W. G. Evolution of neck vertebral shape and neck retraction at the transition to modern turtles: an integrated geometric morphometric approach. *Syst. Biol.* **64,** 187–204 (2015).
9. Werneburg, I. *et al.* Modeling neck mobility in fossil turtles. *J. Exp. Zoolog. B Mol. Dev. Evol.* **324,** 230–243 (2015).
