# Supplementary material for: A Jurassic stem pleurodire sheds light on the functional origin of neck retraction in turtles
Source: Sci Rep. 2017 Feb 16;7:42376. doi: 10.1038/srep42376 (PMC5312562; doi:10.1038/srep42376)
Supplement: Supplementary data 2 [file srep42376-s2.doc]

xread

255 113

Sphenodon_punctatus 0000100101000-0----0-00-000-10100-00?----00000?00----0-00100-0000000---00-0100000-1000-???0--0-1114?1---------------000--0----------------------------------------------------------010-00--------0-00----100000--00--??0?1-1-10--00110000100----------0-0--00[2 3]

Simosaurus_gaillardoti 0110?01111100-0--?-1000-010000100-000----00100000----0-0212--1020-01--001-01-1100??---????????-?????1---------------000--0----------------------------------------------------------0?00?0--------?-00----1???00--00--?????-0??1--0??1????????---------0-0--00?

Owenetta_kitchingorum 000100010102?-0--?0000000000?0000000?----0000??0000000-0?000-000000?-0-00-?-0?00000000????0--0-1114?1---------------010--0----------------------------------------------------------0??-??--------?-0-----1???00--00--??0?1-???0--00?1????????---------0-0--00[2 3]

Anthodon_serrarius 0000??0000020-0--?0000000000000000000----00000000000-0-?0010-0000000-0-000010?00000000????0--0-1114?1---------------010--0----------------------------------------------------------??????--------?-0-----100000--00--01--0-1-10--00?1?00???0?---------?-0--00[2 3]

Odontochelys_semitestacea 0000??01?000??0?-?0?00??0?000?00??00?-0--??0????0000?0-0?000-00?0??002-00-?00???????00????0-??-?????????????????????010--0-----00-------------100--0??0000?-0-0--00?????????????????000?00--------?00?----100000--01-0??0010100?--???0100???01000010?--000--000

Proganochelys_quenstedti 0000?00000000-0--?0000000000000000000001-0000010000000-0?000-000000000-00-00010000000000?00--0?000000011110-011100001100010-00??000000??-000-?000--0000000?00?00?0000000010000000???010000--------00000000000000-001000000001000--00?0000000000?00000000001?000

Proterochersis_robusta ???????????????????????????????????????????????????????????????????????????????????????????????????????????????????????0010-?????00??0??-110??000--0010000?00?00?100000000000000000-???????????????????????????????10??????1?0?1??00??????????000000000?0????0?

Palaeochersis_talampayensis 0000?00100000-1--?010000000000000?00??00-0?1?0?0110000-0?110-000000000000-000?1110?000????0--0???0??????????????????1100010-?0????0??0??-?????000--0010000?00?00?0000???0???????0???110000--------000000?0100000--01000?00121000???0?0000???0000000000000?0?000

Australochelys_africanus ???????10??0??1--?0?00?00?000??0??00?001-?0??01?110000-0??10-00000?00?00?-????1110?00?????0--0???0??????????????????11??????????????????????????????????????????????????????????????????????????????????????????????????????????????????????????????????????000

Kayentachelys_aprix 0010001111000-0--?010000000000?10?000001-1010000120110-00010-000000002000-001010110100?1000--0?000000???????????????1110010-00000[0 1]100000021000100--0010000?000000000000010000000000-0?0?00--------??0000001?0000--111?0?00?01001--00?1?00?????00000000000?20000

Condorchelys_antiqua ??????????????0?-?0???????000???????????????????120?10-0?110-00?0?000[0 2]0??-?01?1011?100????0--0?0000??????????????????1?0010-????0?1?0001???00??10--001000??????????0???????0?0??????010?00?-??----0?00?0??1?00?0--11??0000?0100????0?1?????????0?00000000???000

Heckerochelys_romani 0??00???1100??0?-?01????0??0???1?????????1?1????12??1??0?110-00?0?100200?-??1?10120?00????11-0?0000????????????????????0010-00000?10?0010?10?0110--1100001?000000000000010000000000-?????0--------???????0?????0--?11??????0????--???1????????000000000?0?00000

Eileanchelys_waldmani 00000?111100??0?-10100000?0000?100000?0??1?1000?1202?0-0?1??-00????00?00?-?0001012?100??0011-0-?????????????????????1100?10-??0001?0000102100???0--1??0000??0?0??000?00???00?0???0??????????????????????????????????1?????????????????????????000000010?0?20000

Indochelys_spatulata ???????????????????????????????????????????????????????????????????????????????????????????????????????????????????????0010-??000??00001?2100???0--0??0000??0?0??000????1??????????????????????????????????????????1??????????????????????????010000000?0?00???

Niolamia_argentina 000000011000??0?010100010120?0110?000001-10101102200?100[0 1]12101000?000111100010101??0001??01??0-0101?00000[0 1]00[0 1]000[0 1]1101100?10-??????????????????????????????????????????????????????????????????????????????0????101??????????????????????????????????????0???01?

Ninjemys_oweni 0???????1?????0???0?0?????20???1??00?00211?1011??2?????????????0???????1?0??????????????????????????011000000011001111????????????????????????????????????????????????????????????????????????????????????0????101?????????????????????????????????????????????

Warkalania_carinaminor ??????????????????0??????????????????????????????20031??????????????????????????????????????????????011101--011????????????????????????????????????????????????????????????????????????????????????????????????????????????????????????????????????????????????

Meiolania_platyceps 0000000110001-000101000101200011000000021101011022003100012111000[0 1]0001111000101012000011001[0 1]?0-0101001111111011110111100010-000??10000??0210?1110--1010000?0?1?0000000001000????????01000111200000000000?00122011111100000001001--00??000??0001?0?0000000000111

Chubutemys_copelloi ???0000110001100??0?000?00?00?11??????0??111000?120211?0?112-000000000?1?-?00?10120000????11-0??????????????????????11?0?1?????????0????????????????????????????????????????????????????1?1?????????0????0????????11????????????????????????????000000??0?00010

Mongolochelys_efremovi 0010000110001?000?010000011000110000000211010110120211000112-00000[0 1]000111000111012?001?10011-0-00100000001--011-00001100010-000001?00000021010110--1010001?000000000000010000000000-0000011111000000000000111200--1111000010?0?1--00??100?10001[0 1]000000000?20010

Kallokibotion_bajazidi 00?0??011?0010?00?0100000?000?100?00??0??111?1?0220231001112-1??0??0011100?0111?1?0000?10011-0?0010?0???????????????1100010-00001?000001??100??00--1?10000??001?200000001000?000000-?00000--------0?0??????0--00--?1110000001001--00??????????0000000?000?0?010

Peligrochelys ???0?????00?????01????????1?0??????????1-????????20?1100[0 1]?22-0?????0??11???0101012?001110011-0-0?1??0???????????000011??????????????????????????????????????????????????????????????????????????????????????????????????????????????????????????????????????0?0

Patagoniaemys_gasparinae ???????????????????????????????????????????????????????????????????????????????????????????????????????????????????????0010-000?0??0?00?0?10??110--????????0???0???0???????0?00?????010?01112100000?0?1??0112100--?1???????0?0?1--00?1?????????????????0?02???0

Otwayemys_cunicularius ??????????????????[0 1]????????????????????0-??????????2?1???????????????????-???????????????0????????????????????????????0001???0????00?00??2?0??110--1???00???0?202000000000000001?00-00??011??10???000?1??0?12?0????1??????????????????????????0?0?00??0?0??????

Platychelys_oberndorferi ???????????????????????????????????????????????????????????????????????????????????????????????????????????????????????0010-100001100000011000010--1110000?10111110001001100000001--110??11??1010111000010?1???0--?1???????11?110000??????????100000000?0?00???

Caribemys_oxfordiensis ???????????????????????????????????????????????????????????????????????????????????????????????????????????????????????001??10000?0??00??2????010--11?0000?101111?00010011000000?1--1????1????----??0??0???????0--?1????0?11100?10?0????????????0000??0?0??????

Notoemys_laticentralis ????????????????-?????????000???????????????????3202?0-0??2??0?????00?0???01001?02?000?10011-0?1113????????????????????0010-100001000001021000010--11?0000?1011?010001001?00000001--110?11?-------1?0?201??????0--?1????0011????11?0??????1???100000000?0?00002

Prochelidella_cerrobarcinae ???????????????????????????????????????????????????????????????????????????????????????????????????????????????????????0010-??1011001000021110100--11100001?0?1??10001001000000001--???????????????????????????????????????????????0??????????000000000?0010???

Elseya_dentata 0110111111001100-?0101--100001110000110201011000320230-02122-01000000-010-010010020000110111-??111301---------------110001??10?0-1?1200?1211-0000--1110000?10021010001001?00?00001--10?0111131000010?0201011??10--1122??0??1???11100?110011???0-000000010000002

Myuchelys_latisternum 0110111111001100-10101--1000011100001100-1011000320230-02122-01000000-010-010010020100110111-0?111301---------------110001??1010-1?120001211-0000--1110000110021110001001000000001--1000111131000010002010111110--1122?00011001111000110011???0-000000010000002

Chelodina_colliei 0110111111111100-12101--1000011100001100-101?000320230-12122-01000000-0000110010020000110111-2?111301---------------11100110101001?120000211-0000--1110000110120110001011000000001--1000111131000010102010111110--112211001100111100011001100?00000000010000002

Chelodina_longicollis 0110111111111100-12101--1000011100001100-1011000320230-12122-01000000-0000110010020000110111-2?111301---------------111001101010-11120000211-0000--1110000110120110001011000000001--1000111131000010102010111110--11221100110011110001100110000-000000010000002

Yaminuechelys_maior 0?1?????1100??0?-?2??????1000?1????0??0??????????????0-???22-?????000-0000?10?100??000????11-??1113?1---------------11100110100011?01000021110110--1110000110111010001001000000001--100?1111310000101020101????0--11?2??0??1????01???1????????000000000?0020002

Phrynops_geoffroanus 0110111111001100-12101--1000011100001100-1011000320230-02122-01000000-0000110010020000110111-2?111301---------------1110010-101011101000021110000--1110000110121010001001000000001--1000111131000010002010111110--112210001100111100011001100000000001010000002

Chelus_fimbriatus 1--0101111001100-12101--1000011110000100-1010000320230-02122-01000000-0000110010020000110111-2-111301---------------1110210-101001100100021110000--1110000110121111001020100100001--1011111131000010102010111110--112210001100111100011001100000000000010000002

Araripemys_barretoi 1--1??011101110?-1211000100001110000?000-?11??0?3212?0-02122-01010000-0???01001002??00??0111-0-1113?????????????????11102112101011001100121110110--11110111101201100011---00000101--100?111100000010102??0111110--11221?0011?????????11001100011000110011020002

Erymnochelys_madagascariensis 1--1110111011100-?010000100001110000010202111-00321230-12122-01000001-0100010?10020000110?11-?-111-00---------------1100010-101011101000121110000--1110000110011010001001100100001--100011110000001000201011??10--112???0??100111100?110011???00000000010000002

Pelomedusa_subrufa 1--1110111011100-1210000100001110000010202111-00321230-[0 1]2122-01000000-0100010010020000110111-0-111300---------------1100010-101011101000121110000--1110000110111010001001000000001--1000111100000010002010111110--112210001100111100011001100000000000010000002

Podocnemis_expansa 1--1110111011100-1010000100001110000110212111000321230-12122-01001001-0100010010020000110111-2?111-00---------------1100010-101011101000121110000--1110000110111010001001000100001--1000111100000010002010111110--112211001100111100011001100000000000010000002

Dorsetochelys_delairi 0010001111001?0???01000000000?110?000100-10100??220211?01122-100001002000-000?1012?100?10?11-0?1110?1---------------11??????????????????????????????????????????????????????????????????????????????????????????????????????????????????????????000?????0???010

Pleurosternon_bullockii 0110??1111011100??01000000000?110000???0-1?10??0220211?01112-?00101?020?0?0???10120000?10??????001011---------------11?00111?00011?00000121011000--1??0000??01010000010010000000000-00???0--------??0?00???????????1???????0???1--00??????????000000000?0?00010

Glyptops_plicatulus 01100?11110?11?0??0100?0??00??110?01?001-1?1??00220211?01112-10010100200?-?01?10220000010011-2?0010?1---------------11000111100011?00001021011000--11100001101010000000010000000000-?00010--------??0?001010???0--1112000010???1--00?1????????000000000?0?10010

Dinochelys_whitei 0?10??11110???00??0100000?00???10?01?????????????20?11?????2-???????0?0????????0??0????10???????????????????????????11000111??0011?00001021011000--1110000??000?0000000010000000[0 1]1--00???0--------????0?1?1????????11??????0??????????10011???000000000?0?10???

Neurankylus_eximius ???0??11?10?11?00?1??????000?01?????????????????220?11002122-10?0??00?000001001?220000010011-??11121????????????????11?00111100011?00001021111000--1110000?10?01??00000010000000000-??????????????????101??????????1???????0????--????????????000000000?0?00010

Trinitichelys_hiatti 0010002111011100??110000000000110000?100-101?0002202110?2122-1000010020000010?10220000010011-0?111211---------------1100011110?01??0?0??02111?000--1110000?10?01??00000011000000?00-??00?0--------??????10?????????1???????0??????????????????000000000?0???010

Plesiobaena_antiqua 00100021110111000?1100000000001100000100-1010000220211002122-1000010020000011?10220000010011-??111211---------------11000111100011?0000?021110000--1110000?10?01?00000021?000000100-0000?11????????0??101010??00--?1???????0???1--0??110011???00000000000000010

Boremys_pulchra 00100021110111000?110000000000110000?1???101?0?0220211002122-10001?00?000001?01022000001001????111211---------------11000111100011?00001221110000--1110000?10?01??0000021?000000?00-010010--------????1010112100--?12??????0????--????????????00000000000000010

Baena_arenosa ???00021110011000?0100000000001100000001-1010000220211002122-100001002000-011?10220000010?11-??111210???????????????11000111101011?00000221110000--1110000?10111?000000211000000100-?10?10--------000?1010112200--11220000101001--0011????????000000000?0???010

Chisternon_undatum 0010002111001100??1100000000001100000101-1010000220211002122-1000110020000011?10220000010011-0?111211---------------110001??100011?0000?2211-?000--1110000?10?01?00000021100000010???000?1112?0000??0?101011??00-??1?2?000101001--0011????????000000000?0?00010

Portlandemys_macdowelli 00110001110?10010?1??????000001100000101-1010000220211001122-1000011020???010010120000010011-0?111100???????????????110?????????????????????????????????????????????????????????????????????????????????????????????????????????????????????????0???????????113

Plesiochelys_etalloni 00110001110[0 1]10010?1100000000?01100000101-1010000220211001122-101001002010001?0101200000100?????111100???????????????1100010?100011?00001221010[0 1][0 1]0--1110000?10?210000[0 1]00010000000[0 1]00-??????????????????0010????????11????0010???1--0??110011???000000000?0?1[0 1]113

Solnhofia_parsonsi 00110001110[0 1]10000?110000?00000110000-110-1110--0220211001122-1000001010100011010220000010011-0?111100???????????????1100010-??0011?0000202101?110--???0011?0002000000??????000?00???110?00--------??????1?10??????11?2??0010?0??--???11001100000000000?10011113

Thalassemys_moseri 00100011110011010?1100000000001100000?01-1110000220211001122-1010-?102000001?11022000?010011-0?111100???????????????11?001??10001??0?0???2?0???10--????????1?02????00??????0?0?0????11??00--------0?0?1????????????1????????????????????????????0000000?0?10113

Santanachelys_gaffneyi 0010000111011000??0100000000?0110?00?101-111000022021100?122-1010-?002010?01??102??00?010?1?????????0???????????????110001???00011?00100?2100?110--1110010?00120?00?0???????????????11???????????0????0?1?1???????11?2?????0????--0??111011?1?000000000???0[0 1]113

Toxochelys_latiremys 1011000111011?00011100000000101100000100-101000022021100?122-1000011020100010010220010010011-0?1111??---------------1100010-200011000101021110110--111001110022010100???1??0???010??11?10111200??0?1???01011??00--11???????00???--0??111011?1?[0 1]00000000111[1 2][0 1]113

Caretta_caretta 1--100011[0 1]00120001010000000010110100-110-1011000220111101122-1020-11010100011010221010010011-0?11?100---------------1100010-200010000101021110110--1110011100220?010001---10?00000??1111011120001111002011111110--1122111-100111--00011201101010000000011121113

Chelonia_mydas 1--1?0011100120001010000000010110101-11201011000220111001122-1020-11010100011010221010010011-0?111100---------------1100010-200011000101021110110--1110011100220?010001---10?0?000??1111011120001111012011111110--1122111-100111--00011201101?1000000001112?113

Mesodermochelys_undulatus ???????????????????????????????????????????????????????????????????????10?????????????????????????????????????????????01010-200011000100?2???-110--1110011?00?20?011----------------1110011120000001002010111110--1122111-101111--0001?20???1?1000000?011020???

Dermochelys_coriacea 1--1010110000-00-10000000000101100000001-1010000220110-02122-1020-00010100010010221000010011-0?111201---------------110201-?20-0-300-1---2----11-??1110011?0??20?011----------------11110111200011010020-0111110--1122111-101111--000112011?1?1-0000--?11100113

Macroclemys_schmidti 1??1??011001110?0121000000000?110?010100-101000?220241001122-1000?110201000100102??000?100101001111?0?????????????????????????????????????????????????????????????????????????????????????????????????????????????????????????????????????????????????0?????1?3

Macroclemys_temminckii 1--1000110011100012100000000001100010100-1010000220221001122-100?111020100010010220000010010100111100???????????????1100010-120001000000011110110--11100111002201000001-1-00?001100-1111011120010111012011112200--11221000100001--00111001110?10000000001121113

Protochelydra_zangerli 1--10?0110?11100???100000000?0110000?????101????220?21???122-10001????010?0???10???0??010??????????0????????????????11???1???????????0???????0100--11100?1?0??20?0000?1-1-00?0?110?????????????????????????????????12??????0???1--0???????????000000000?????113

Chelydra_serpentina 1--10001100111000?2100000000001100010100-1010000220221001122-1000111020100010010220000010010??0111100???????????????1100010-120001000002021110110--1110011?002201000001-1-0010?1100-1111011120010111012011111200--11221000100011--00?110011???10000000001101113

Platysternon_megacephalum 1--1000110011100010100100000001100010100-1010000220221001122-1000111020100010010221000010010100111101---------------1100010-100001100001021110100--11100001002202100001-1-000000000-1101011121010111002011111200--11220000100011--00111001100?00000000000121113

Mongolemys_elegans 1--[0 1]000111011100??210000000000110000000001010000220211001122-100001002010??0001022000001001??0?011101---------------1100010-10?0?10000??021110000--1110000?100210100001-1-00?000000-?????11121010111012?111?1100--112??????0???1--0??1100?????00000000010100111

Gopherus_polyphemus 1--100011101110011210000100000110000000201010110220221001122-1000110010100010010220000010010100111101---------------1100010-101001000011021110000--11100000100210100001-1-00001000101111011121010111012011111110--11220001101011--00110001110000000000010100113

Eurotestudo_hermanni 1--10001110111001?210000100000110000000201010110220221001122-10001100[0 1]0100010110220000010010100111101---------------1100010-101001000011021110000--11100000100200100001-1-00001000101111011121010111012011111110--11221001101011--00110001110000000000010100113

Chelonoidis_gringorum ??????????????????????????????????????????????????????????????????????0????????????????????????????????????????????????0010-??1001000011101110000--11100000?0?2??100001-1-0000100010??????????????????????????????????1?0?101?????????????????00000000010100???

Chelonoidis_chilensis 1--100011101110011210000100000110000000201010110220221001122-10001100?0100010010220000010010100111101---------------1100010-101001000011121100000--1110000000021?100001-1-00001000101101011121010111012011111110--1122[0 1]001101011--000100011100000000000?0?00113

Stylemys_nebraskensis 1--1??0111011?0???21000010000?11??????0????1?????202?1?0?122-?0???1?01????????10????0?????????01111?1???????????????11?0010-?0?0?1000001021110000--1110000110122?100001-1-0000100010??????????????????????????????11?2100111100?--??1100011000000000000?0?00113

Chrysemys_picta 1--100011101110011210000100000110000000201010000220211001122-0000110020100010110220000010010100111101---------------1100010-101011000000021110000--11100001101210100001-1-00001000101111011121110111012011111110--11221001101011--00111001100000000000010100113

Trachemys_scripta 1--100011100110011210000100000110000000211010000220211001122-1000110020100010110220000010010100111101---------------1100010-101011000010021110000--11100001101210100001-1-000000001011?-0--1211101110?2011--??-0-----21001101011--0011-----00000000000010100113

Emys_orbicularis 1--100011001110011210000?000001100000?00-1010000220211001122-00001100[1 2]0100010110220000010010100111101---------------1100010-1010110000000211101010010100001001201000001-1-0010000???111101112101011101201111111???11221001101?11--00?11001100000000000010100113

Geoclemys_hamiltonii 1--1000110011100112110001000001100000000-1010110220211001122-10001110[1 2]0100010110220000010010100111101---------------1100110-101011100001021110000--11100000101211100001-1-00101000101111011121010111012011111110--11221001101011--00111001100000000000010100113

Echmatemys_wyomingensis ???????????????????????????????????????????????????????????????????????????????????????????????????????????????????????0010-101011?0000?021110000--11100001100211100001-1-0010000010??????????????????201?????????112??????0???1--?0??????????000000000?0?00113

Emarginachelys_cretacea 1--1000111?111000??100001000?0110000?????1010??1220211??1122-100011????10?01??102200??010???????????1---------------11?011??12?0?1?000???21110100--11100?0?0?120?000001-1-00????00??11???111????????????1?????????11?2?????0???1--01?110011???00000000001?00113

Baptemys_wyomingensis 1--10001110111000?2100001000001100000002?1010001220211001122-1000[0 1]?002?10001?01022?00?0-2-1??0?111101---------------1100110-100011?0000?021110000--1110000110121?000001-1-001100000-11?1?111??????????2?1111??1???1122?????01??1--1??110011???000000000?0?00113

Dermatemys_mawii 1--100011[0 1]0111000?210000100000110000000201010001220211001122-10001100201000110102200000-2-1010?111101---------------1100010-1[0 1]0011101000021110000--1110000?101200100001-1-100100000011110110-001011101201111??10--11221001101101--00?11001100000000000010100113

Xenochelys_formosa 1--10?01100?1100112??????00????10?010000-1?10?0?2????1?01122-?020-???20??????0?????00??110?????1111?1---------------11?0110-1?1012101000021110000--11100001002201100001-1-00-1-20011??????????????????????????????????????????????????????????000000000?0?10113

Staurotypus_triporcatus 1--100011001110011211010100000110001?000-1010001220211101122-1000100020100010010220000011010101111101---------------1100210-11001210[0 1]0000211100010111100101002201000001-1-00-1-2001111110111100101110121-1111?10--11221001100101--110110011???00000000011100113

Sternotherus_odoratus 1--1000110011100112100101000001100000000-1010001220211101122-1000110020100010010220000011010101111101---------------1100110-11001211100002111000101---?1--1002201000001-1-11-1-2001111110111100101110121-1111110--11221001101101--11011001100000000000011100113

Kinosternon_flavescens 1--1000110011100112100101000001100000000-1010001220211101122-100010002010001001022000001101??01111101---------------1100110-11001201100002111000111---?1--1002201000001-1-11-1-200111111011110???1110121-1111110--11221001101111--11011001100?00000000010100113

Basilemys_variolosa 1--1??0111?1??00???10000100?00110?00???????1?????20?21???122-?0???????????????10????????????????????????????????????11000112101111?0[0 1]000021111000--1110000100020?000100[0 2]11001000001011?1?1?0-0?????????????????????12??????01011--00?1000??0000?0000000?0120111

Yehguia_tatsuensis ???????????????????????????????????????????????????????????????????????????????????????????????????????????????????????00112????11???000?2???1000--1110000??0?2??000100010000000000-??????????????????????????????11???????0????????11????????00000001010??????

Adocus_beatus 1--100011101110011210000100100110000010211010001220211101122-100011102?10101?01022000?010011-1?011100???????????????11000112101111?0[0 1]00?021111000--11100001000200000100010001010000-1111?100-0010111012?111???????11220001101011--011110011???000000000?0100111

Hoplochelys_crassa ???????????????????????????????????????????????????????????????????????????????????????????????????????????????????????0210-11?0?1?000??021110000--1110000000020?000001-1-00-1-1000-??????????????????201?????????1122??????????--????????????000000000?0??????

Apalone_spinifera 1--1010111011100112110001001001112101001-1111001220221101122-1001111010101010010220000010011-12111101---------------11020112100013001-03------110--111101-100-20-0-1----1-----------11010120-001011100201-111110--11221101100111--000110111001[0 1]00000--01-101113

Plastomenus_aff._thomassii 1--10?011101110?11211000100?0?11----1000-111100?220221101122-100111101010101001022?000??00101121111?1---------------11020112100013001-03------100--?????101-0-2--??1----------------??????????????????20??1???????????????????????????????????100000--0?-?01113

Pelodiscus_sinensis 1--10101110111001121100010010011?2??1000-1111001220221101122-1001011010101010010220000010011-1?1111?1---------------11?20112100013001-03------110--11110101-0-2--001----------------110?0120-00?01110020--11??10--?1????0??????????????????001100000--01-101113

Lissemys_punctata 1--1010111011100112110001001001112101000-1111001220221101122-1001101010101010010220000010010012111101---------------11020112100013001-03------110--111101-101-20-0-1----1-----------11010120-001011100201-11--10--11221101100111--010110111001100000--01-101113

Shachemys_laosiana 1--1??011101??0?11[1 2]??????00???11????????????????2????????122-10001??0?0????10110???000?1?0?????1111?????????????????11?0010-??10-1?[0 1]20001210-0100--1110000?000201000000010001000000-??????????????????????1???????????????????????????????????000000000?0?0?113

Anosteira_ornata 1--1??0111?1??001??10000100??011?200?????1111???220221???122-1001[0 1]1?0?010?01??1022?0??010??????????01---------------11?101121000120010030211?-100--11100101002201001----1-----------???????????????????????????????1????????????--???110211???000000000?-?11113

Carettochelys_insculpta 1--1010111011100112100101001001112000000-1111001220221101122-1001111010101010010220000010011-1?111101---------------11010112100012001000-2----100--11100101002201001----1-----------11010110-0010111002011111110--1122001-100111--010110211000000000-001-101113

Xinjiangchelys_wusu 0110?0111100??0?01210000000000110?000?-??1?1?00?22021100?112-000001102010000001022?00??100?1-0?0111?0011?????0??00??1100010-??001110[0 1]001021111100--1?100001000200000000010000000100-0[0 1]?????-------1?0??????????????1??0?001?????--????1001100000000001000?10111

Annemys_levensis 00?1??01110?11000121000000000011000000-0-1?10000220211001112-000001?02010000001022?00??100?1-0?011?00011?????0?0000?1100010-100011101001021101100--11100001000200000100010000000100-??????????????????0?00????????1112000010???1--?0??????????000000010?0?20111

Annemys_latiens 01?0??11110?110001210?0??0????11???????????????02???11001112-00?0???0?0?0???00102??00??100??-??011??????????????????11?0010-100011?0[0 1]0010211[0 1]1100--11100001000200000100010000000100-???????????????????????????????1??000010????--????????????000000010?0?20111

Xinjiangchelys_radiplicatoides 01?0??11110?110001[1 2]?0????0????11??0????????????0220?11001112-00?????0?0?000000102??00?0100?1-0?0112?????????????????11?0010-1000?1?0000102111??00--1?100001000200000100010000000100-??????????????????0???????????111200001?????--????????????000000010?0?10111

Xinjiangchelys_junggarensis ???????????????????????????????????????????????????????????????????????????????????????????????????????????????????????001??100011100001021111100--11100001000200000100010000000100-?111?11??????011??000011??00--1112?????0???1--0??1????????000000010?0010???

Chengyuchelys ???????????????????????????????????????????????????????????????????????????????????????????????????????????????????????0010-?000???0000?221101100--1?10000?0000????0000000000000?0????????????????????????????????????????????????????????????00?000010?0?00???

Hangaiemys_hoburensis 0011000111001100011100000000001100000000-101000022?211001112-[0 1]00001002010000?01022010001001010?0111?0???????????????1100010-100011?0000?021110100--1110011100020?000001-1?000000[0 1]00-?111?111200000110?2?10?1??????11220?0010???1??????????????000000010?1100111

Judithemys_sukhanovi ???1??01110?1100??110000?0000011???0??0??101?00?220211?0?112-1000011020100000?10220100010011-0?0111?????????????????1100010?100011?00001021110100--1110011?000200000001-1?000000[0 1]00-?111011120000011012?10111210--1122000010???1--00?11001100?00000000001100111

Changmachelys_bohlini ???1??011100??0??111000000000011????0000-???????22021??0?1???100?01?020100?0?0102??10001001??0??1?1?????????????????1100010-?00011?0000?0211??1?0--1?1001??00?20000??????????????????111?1?1?0??0?1?0?????1????0--11?2??0?10???1--????10011000?0000001?01?10111

Dracochelys_bicuspis ????0???110?110001??0000?000??1100000001-101000?220[1 2]11001112-1000011020?0?010?1022?00001001??0?0111?1---------------110001??100011?000011?111?110--???0011?000200000????????????????0111?110-0000011000010?????0--11?20?0010???????????00?????010111101?1021111

Ordosemys_leios 0??????1????????0?[1 2]????0????????????0000-????????2??111???1????0?0???????????0?????10??100???0?0?1?????????????????????0010?100011100001021010110--???00???0002000000??????0?0?0[0 1]00-011?0111210000?10?0??01112?0--11?20?001000?1??00?1????????1000000100101[0 1]???

Ordosemys_skull 00100001110?1100??110000?000?01100000?-0?1010000220211?01112-1000011020?0?00?01022?100010?11-0?0111?????????????????11????0-???0????????????????????????????????????????1???????????????????????????0???????????????????????????--??????????????????????????111

Sinemys_brevispinus ????????????1???01?????0?000??1????0????????????2212?100?11?-0?????00?0?0?00?01022?100?1001??0?01?1????????????????????0011??00?11?0?00?1211?0110--111001010002000000???1??0?0001???11???1?0-000001?0?0????112????1122?10010??????????????????1?100010101?21111

Sinemys_lens 0010??111101110001210?00?0000?11??00??-0-??1????22?2?100?1?2-00000?002010??0001022?10001001??0?0111?1---------------110001??1000111000011211101[0 1]0--111001010002000000???1??0?000?0????????????????????0???1????????1???????0??????????1001100001100010101?21111

Sinemys_gamera 0?10??111101110?01210?00000?0011????00-0-???????221221001112-0000?1002010?00?01022?10001001010?0111?1---------------11?0011?????1?10000??2?11?1????????????????00??0???????????0??????????????????????????????????????00001????????????????????1?0?0?01?????111

Liaochelys_jianchangensis 0011?0011101??0?01210???0000??110?0??????????????????10????????0???????100???010???????100???????????????????????????100010???0?11?01001021010110--???0011?00?20?000????????????????011??1????????1?0?????1112?0--???????????????????????11?0?1001110[0 1]101?1[0 1]???

Xiaochelys_ningchengensis_ ???1??01110???0?01[1 2]10?0?0000??110?0??????????????????1?????????????????100???010???????100???????????????????????????1?0010-??0011?00001021?1010????????????0?200??0???????????0?0???1???1?12?????1?0?????111200--?????????????????????????????000000110??10???

Manchurochelys_manchoukuoensis ???1?00?1101??0?01210?0??0000?110??00?-??101?0??2????100?112-10?00?1020100?0?010???100?100?1-0?0111?1---------------1100010???0?11?000010211101???-??????????????????????????????????11??1?0?0????1?0?????1???00--11?20?0?1??????????????11?0?00100001101?00111

Basilochelys_macrobios ????????????1100012??????????????????????101????2????1101112-00?0??10?0????01010???100?100???0?0111??????????????????1?0011???0111?0?00?021111000--1110000?00[0 1]2??000????1??01???0????11???--------1?0?0??1?????????????????01011--?011????????0?0000010?0?00111

Siamochelys_peninsularis ???????????????????????????????????????????????????????????????????????????????????????????????????????????????????????0011??000111000010211?0100--1?10000??01201000100010000000?00-??????????????????????????????????????????????????????????000000010?0?[1 2]0???

;

cnames

{0 Nasal_A present absent;

{1 Nasal_B nasals_contact_another_medially_along_their_entire_length medial_contact_of_nasals_partially_or_fully_hindered_by_long_anterior_fl;

{2 Nasal_C dorsal_exposure_of_nasal_large greately_reduced_relative_to_that_of_all_other_elements;

{3 Prefrontal_A medial_contact_on_dorsal_skull_roof_absent medial_contact_on_dorsal_skull_roof_present;

{4 Prefrontal_B prefrontal-vomer_contact_present prefrontal-vomer_contact_absent;

{5 Prefrontal_C prefrontal-palatine_contact_present prefrontal-palatine_contact_absent;

{6 Prefrontal_D prefrontal_exposure_large reduced absent_or_near_absent;

{7 Prefrontal_E prefrontal_heavily_sculptured_present absent;

{8 Lacrimal_A present absent;

{9 Frontal_A frontal_contribution_to_orbit_absent present;

{10 Frontal_B* not_fused fused;

{11 Parietal_A parietal-squamosal_contact_present absent;

{12 Parietal_B parietal_contact_with_pt,_epipt,_and/or_palatine_absent present;

{13 Parietal_C lenght_of_anterior_extension_of_the_lateral_braincase_wall_inter elongated short,_enclosing_the_foramen_nervi_trigemini;

{14 Parietal_D overhanging_process_of_the_skull_roof_absent present;

{15 Parietal_E processus_inferior_parietalis_forming_posterior_margin_for_nerv_trigemini_absent ..._present;

{16 Parietal_F* not_contribute_to_the_processus_trochlearis_oticum contributes_to_the_processus_trochlearis_oticum;

{17 Parietal_G* forming_part_of_the_foramen_stapedio-temporalis not_forming;

{18 Parietal_H* absent_or_weak,_foramen_stapedio-temporale_concealed_in_dorsal_view moderate,_f.s.t._but_not_entire_processes_trochlearis_exposed_in_dorsal_view strong,_entire_processus_trochlearis_exposed_in_dorsal_view;

{19 Jugal_A jugal-squamosal_contact_present absent;

{20 Jugal_B jugal_participation_to_upper_temporal_rim_absent present;

{21 Quadratojugal_A present absent,_due_to_the_presence_of_a_deep_lower_temporal_emargination;

{22 Quadratojugal_B quadratojugal-maxilla_contact_absent present;

{23 Quadratojugal_C quadratojugal-squamosal_contact_below_cavum_tympani_absent present;

{24 Squamosal_A squamosal-postorbital_contact_present absent;

{25 Squamosal_B squamosal-supraoccipital_contact_absent present;

{26 Squamosal_C* posterolateral_protuberances_developing_horns_absent small_protuberances big_protuberances_developed_as_horns;

{27 Squamosal_D* long_posterior_process_protruding_beyond_condylus_occipitalis_absent present;

{28 Squamosal_E*_ Qu-Sq_contact_tightly_sutured wide_open;

{29 Postorbital_A postorbital-palatine_contact_absent present,_foramen_palatinum_posterius_situated_posterior_to_the_orbit;

{30 Supratemporal_A present absent;

{31 Premaxilla_A external_nares_divided united;

{32 Premaxilla_B fusion_of_premaxilla_absent present;

{33 Premaxilla_C foramen_praepalatinum_present absent,_premaxillae_well-ossified absent,_foramen_intermaxillaris_present;

{34 Premaxilla_D exclusion_of_premaxilla_from_the_apertura_narium_externa_absent present;

{35 Premaxilla_E distinct,_medial_premaxillary_hook_along_the_labial_margin_absent present;

{36 Maxilla_A* do_not_contact_each_other_in_ventral_view contacts_each_other_in_ventral_view;

{37 Maxilla_B* Upper_triturating_surface_not_involving_palatine_or_its_contribution_is_minor involving_palatine;

{38 Maxilla_C*_ Secondary_palate_formed_by_premaxilla,_maxilla,_and_vomer,_palatines_not_contacting_in_midline_absent formed_by_premaxilla,_maxilla,_and_vomer,_palatines_not_contacting_in_midline_present;

{39 Maxilla_D*_ Triturating_surface_with_only_labial_ridge_present labial_and_lingual_ridge_present labial,_lingual_and_accesory_ridges_present;

{40 Maxilla_E*_ Accesory_ridge_on_maxilla_present_all_along_the_triturating_surface accessory_ridge_only_in_some_sectors_of_the_triturating_surface;

{41 Vomer_A paired single single,_greatly_reduced;

{42 Vomer_B vomer-pterygoid_contact_in_palatal_view_present absent,_medial_contact_of_palatines_present;

{43 Vomer_C vomerine_and_palatine_teeth_present absent;

{44 Vomer_D vomer-premaxilla_contact_present absent;

{45 Vomer_E*_ Narrow_and_tall_ventral_crest_on_vomer_absent present_all_along_the_vomer;

{46 Vomer_F*_ Domed_palate_absent present;

{47 Palatine_A palatine_contribution_to_anterior_extension_of_lat_braincase_absent present,_well-developed;

{48 Quadrate_A flooring_of_the_cranioquadrate_space_absent by_pt,_but_pt_does_not_cover_the_prootic by_pt by_qu_and_pro;

{49 Quadrate_B_+_C development_of_the_c.t._shallow,_but_not_developed_antpost shallow,_but_anteroposteriorly_developed deep_and_anteroposteriorly_developed;

{50 Quadrate_D precolumellar_fossa_absent large_and_deep;

{51 Antrum_postoticum_A antrum_postoticum_absent incipient fully_developed;

{52 Quadrate_F:_incisura_columella_auris present,_but_qu_and_the_op_for_an_angle_of_90_degrees_in_lat_view present,_but_qu_and_the_op_for_an_angle_less_90_degrees_in_lat_view present_and_closed,_but_only_enclosing_the_stapes present_and_closed,_enclosing_stapes_and_the_Eustachian_tube partially_closed,_allowing_see_the_columella_auris_in_posterior_view;

{53 Quadrate_G processus_trochlearis_oticum_absent present;

{54 Quadrate_H*_ Processus_trochlearis_oticum_formed_by_a_great_contribution_of_quadrate small_contribution_of_the_quadrate;

{55 Quadrate_I* Quadrate-basisphenoid_contact_absent present;

{56 Epipterygoid_A present,_rod-like present,_laminar absent;

{57 Pterygoid_A pterygoid_teeth_present absent;

{58 Pterygoid_B basipt_process_present_and_movable_articulation basipt_process_present_and_sutured_articulation basipt_process_absent_and_sutured_articulation;

{59 Pterygoid_C triangular_in_shape reduced_to_an_interpterygoid_slit reduced_to_a_paired_foramen_caroticum_laterale;

{60 Pterygoid_C2* Intrapterygoid_slit_extensive,_completely_covering_fcb_no yes;

{61 Pterygoid_D pterygoid-basioccipital_contact_absent present;

{62 Pterygoid_E processus_trochlearis_pterygoidei_absent present;

{63 Pterygoid_F foramen_palatinum_posterius_present present,_but_open_laterally absent;

{64 Pterygoid_G medial_contact_of_pterygoids_present absent;

{65 Pterygoid_H pterygoid_contribution_to_foramen_palatinum_posterius_present absent;

{66 Pterygoid_I vertical_flange_on_lateral_process_absent vertical_falnge_on_lateral_process_present;

{67 Pterygoid_J* not_reaching_the_exoccipitals reaching_the_exoccipitals;

{68 Pterygoid_K* Fossa_podocnemidoidea_absent present;

{69 Pterygoid_L*_ Processus_pterygoideus_externus_like_in_Proganochelys like_in_testudinoids like_in_Kayentachelys;

{70 Pterygoid_M* Basisphenoid_and_pterygoid_in_the_same_level Basisphenoid_and_pterygoid_in_different_levels,_step_between_both_bones;

{71 Supraoccipital_A crista_occipitalis_poorly_developed protruding_significantly_posterior_to_the_foramen_magnum;

{72 Supraoccipital_B large_supraoccipital_exposure_to_dorsal_skull_roof_absent present;

{73 Supraoccipital_C* horizontal_ventral_crest_in_the_supraoccipital_absent_or_poorly_developed_anteriorly horizontal_ventral_crest_present_along_all_the_crista_supraoccipitalis;

{74 Exoccipital_A medial_contact_of_exoccipitals_dorsal_to_foramen_magnum_absent present;

{75 Basioccipital_A with_two_or_one_ventral_basioccipital_tubercle tubercle_absent;

{76 Basiocccipital_B* Deep_C-shaped_concavity_between_basioccipital_tubera_absent Deep_C-shaped_concavity_present;

{77 Prootic_A* dorsal_exposure_large dorsal_exposure_reduced_or_absent;

{78 Opisthotic_A loosely_articulated tightly_sutured;

{79 Opisthotic_B depressions_for_musculature_absent present;

{80 Opisthotic_C ventral_ridge_on_opisthotic_absent present,_with_an_incipient_enclosed_middle_ear_region present,_but_modified_with_a_enclosed_middle_ear_region;

{81 Opisthotic_D:_processus_interfenestralis present,_robust,_not_reaching_the_floor_of_cavum_a-j present,_robust,_reaching_the_floor_of_cavum_a-j present,_small,_reaching_the_floor_of_cavum_a-j;

{82 Basisphenoid_A rostrum_basisphenoidale_flat rod-like,_thick,_and_rounded;

{83 Basisphenoid_B paired_pits_on_ventral_surface_absent present;

{84 Basisphenoid_C* reduced_to_a_v-shaped_basisphenoid_trapped_between_the_pterygoids_and_the_basioccipital_absent reduced_to_a_v-shaped_basisphenoid_trapped_between_the_pterygoids_and_the_basioccipital_present;

{85 Basisphenoid_E* rough_surface_between_basisphenoid_and_basioccipital_absent present;

{86 Hyomandibular_nerve_A :_path_of_hyomandibular_branch_facial_nerve_through_cranio-quadrate_space_parallel_to_vena_capitis_lateralis independent_to_vena_capitis_lateralis;

{87 Stapedial_artery_A posterior_to_fenestra_ovalis_between_paraoccipital_process_and_qu anterior_to...;

{88 Stapedial_artery_B relatively_large significantly_reduced_in_size absent;

{89 Stapedial_artery_C*_ Foramen_stapedio-temporalis_located_in_the_dorsal_part_of_the_otic_region_and_points_dorsally located_in_the_anterior_wall_of_the_otic_region_and_points_anteriorly;

{90 Recessus_scalae_tympani_A* almost_inexistent,_not_surrounded_by_bone well_developed;

{91 Foramen_jugulare_posterius_A* separated_from_fenestra_postotica coalescent_with_fenestra_postotica;

{92 Foramen_jugulare_posterius_B* separated_from_fenestra_postotica_by_pterygoid separated_by_opisthotic_and_or_exoccipital;

{93 Foramen_nervi_hypoglossi_(XII)* not_covered_ventrally_by_an_extension_of_the_pterygoid_and_the_basioccipital covered_ventrally_by_an_extension_of_the_pterygoid_and_the_basioccipital covered_ventrally_by_an_extension_of_the_bo;

{94 Canalis_caroticum_C Pattern_A Pattern_B Pattern_C;

{95 Canalis_caroticum_D*_ junction_of_palatine_artery_and_internal_carotid_artery_not_enclosed_in_bone enclosed_in_bone;

{96 Canalis_caroticum_E*_ Canalis_carotici_interni_posterior_to_bifurcation_in_ac_and_ap_not_covered_ventrally_by_bone covered_ventrally_by_bone;

{97 Canalis_caroticum_F*_ Arteria_palatina_enters_the_skull_through_the_interpterygoid_vacuity_or_intrapterygoid_slit through_foramen_posterius_canalis_carotici_palatinum_or_split_of_branches_enclosed_in_skull;

{98 Canalis_caroticum_G*_ fpcci_(entrance_of_internal_carotid_artery_into_the_skull)_absent formed_by_pterygoid formed_by_pterygoid_and_basisphenoid formed_by_pro,_pro_and_bs,_or_pro_and_pt formed_by_bs;

{99 Fenestra_perilymphatica_A large relatively_small;

{100 Cranial_scutes_A* present absent;

{101 Cranial_scute_B* Scute_D_meeting_in_midline_no yes;

{102 Cranial_scute_C* Scute_X_much_smaller_than_D_scute_no yes;

{103 Cranial_scute_D* X_scute_partially_separates_G_scales_no yes;

{104 Cranial_scute_E* Scutes_A,_B,_and_C_forming_a_continuous_posterolateral_shelf_yes no;

{105 Cranial_scute_F* D_scute_high low;

{106 Cranial_scute_G* B_scute_a_recurved_horn_no yes;

{107 Cranial_scute_H* B_scute_in_cross_section_triangular round;

{108 Cranial_scute_I* Scute_B_and_D_in_contact_yes no;

{109 Cranial_scute_J* A_scute_small_and_not_forming_a_large_shelf_no yes;

{110 Cranial_scute_K* A_scute_small_A_scute_very_large A_scute_comparable_in_size_to_B_scute;

{111 Cranial_scute_L* Y_and_Z_scutes_relatively_larges_mall large;

{112 Cranial_scute_M* Y_scute_pentagonal_pointing_posteriorly_and_separating_the_medial_contact_of_G_scutes rectangular_not_separating_the_medial_contact_of_G_scutes;

{113 Cranial_scute_N* H_scute_present absent;

{114 Cranial_scute_O* Scale_F_formed_by_several_scales Scale_F_formed_by_only_one_scale;

{115 Cranial_scale_P* Scale_J_formed_by_several_scales Scale_J_formed_by_only_one_scale;

{116 Teeth_A teeth_present_in_premaxilla,_maxilla,_and_dentary teeth_absent__in_premaxilla,_maxilla,_and_dentary;

{117 Upper_temporal_fenestra_A present absent;

{118 Dentary_A medial_contact_of_dentaries_fused sutured_only;

{119 Carapace_A carapacial_scutes_present partially_present absent;

{120 Carapace_B tricarinate_carapace_absent present,_but_only_slightly present_and_pronounced;

{121 Carapace_C absent present;

{122 Carapace_D_* Sculpturing_of_the_shell_absent present;

{123 Carapace_E* Sculpturing_of_the_shell_like_in_Hydromedusa like_in_Pleurosternon like_in_trionychians;

{124 Nuchal_A cervical_articulates_with_nuchal_along_a_blunt_facet articulation_absent cervical_articulates_with_nuchal_along_a_raised_pedestal;

{125 Nuchal_B elongate_costiform_process_of_nuchal_absent present,_process_crosses_peripheral_I_to_contact_pe_II_ present,_contacts_pe_III;

{126 Nuchal_C* wider_than_long longer_than_wide_or_as_long_as_wide;

{127 Neural_A neural_formula_6>4<6<6<6<6_absent present;

{128 Neural_B* irregular_in_shape,_wider_than_long regular,_often_hexagonal,_longer_than_wide;

{129 Peripheral_A more_than_11_pairs 11_pairs 10_pairs less_than_10_pairs;

{130 Musk_ducts_A* absent present;

{131 Costal_A medial_contact_of_costal_I_absent present;

{132 Costal_B medial_contact_of_posterior_costals_absent medial_contact_of_up_to_three_posterior_costals_present medial_contact_of_all_costals_present;

{133 Costal_C absent,_costals_fully_or_almost_fully_ossified,_fontanelles_abs_or_red present;

{134 Costal_D* absence_of_alternative_short_and_long_ends_in_the_lateral_part_of_the_costals presence;

{135 Suprapygal_A* one_element two_elements more_than_2_elements absent;

{136 Cervical_A one_cervical_present cervicals_absent,_carapacial_scutes_otherwise_present more_than_one_cervical_present;

{137 Supramarginal_A complete_row_present partial_row_present absent;

{138 Vertebral_A 4 5;

{139 Vertebral_B vertebral_II-IV_broader_than_pleurals vertebrals_II-IV_narrower_or_as_narrow_as_pleurals;

{140 Vertebral_C sulcus_between_V_3_and_4_on_neural_VI on_neural_V;

{141 Marginal_A* marginal_scales_overlap_onto_costals_absent present;

{142 Plastron_A connection_between_carapace_and_plastron_osseous ligamentous;

{143 Plastron_B central_plastral_fontanella_absent present;

{144 Plastron_C plastral_kinesis_absent present;

{145 Plastral_kinesis_A* anterior anterior_and_posterior;

{146 Plastral_kinesis_B* between_hyo_and_hypoplastron between_hyo_and_epi-entoplastron;

{147 Entoplastron_A anterior_entoplastral_process_present absent;

{148 Entoplastron_B size_of_posterior_entoplastral_process_long short;

{149 Entoplastron_C distinct_posterolateral_entoplastral_process_present absent;

{150 Entoplastron_D entoplastron_V-shaped_absent present;

{151 Entoplastron_E present absent;

{152 Entoplastron_F* entoplastron_tightly_sutured_with_hyoplastron_yes no;

{153 Epiplastron_A epiplastra_and_entoplastron_narrow_and_elongate_absent present;

{154 Epiplastron_B* thick_anterior_border thick_anterior_border_absent;

{155 Hyoplastron_A axillary_buttresses_contact_peripherals_only peripherals_and_first_costal;

{156 Hyo-hypoplastron_A* not_fused fused;

{157 Hyoplastron_B* Axillary_buttress_terminates_on_peripheral_2_or_1 terminates_on_peripheral_3 terminates_on_peripheral_4;

{158 Mesoplastron_A 1_or_2_pairs_of_meso_with_medial_contact 1_reduced_pair absent;

{159 Hypoplastron_A inguinal_buttresses_contact_peripherals_only peripheral_and_costal_V peripherals,_costal_V,_and_costal_VI;

{160 Hypoplastron_B* Inguinal_buttress_terminates_on_peripheral_8 7 6;

{161 Xiphiplastron_A distinct_anal_notch_absent present;

{162 Xiphiplastron_B xiphiplastra_narrow_absent present;

{163 Plastral_scutes_A present absent;

{164 Plastral_scutes_B pronounced_midline_plastral_sulcus_sinuous_absent present;

{165 Gular_A one_pair only_one_scute;

{166 Extragular_A present absent;

{167 Extragular_B medial_contact_of_extragulars_absent present,_contacting_one_another_anterior_to_gulars present,_contacting_one_another_posterior_to_gulars;

{168 Extragular_C anterior_plastral_tuberosities_present absent;

{169 Extragular_D* Only_in_the_epiplastra Reach_the_entoplastron;

{170 Intergular_A absent present;

{171 Humeral_A 1_pair 2_pair_subdivided_by_a_plastral_hinge;

{172 Humeral_B* Humero-pectoral_sulcus_only_in_the_hyoplastra humero-pectoral_sulcus_crossing_the_entoplastron;

{173 Pectoral_A present absent;

{174 Pectoral_B* antero-posteriorly_developed very_short_antero-posteriorly;

{175 Abdominal_A present,_with_medial_contact present,_medial_contact_absent absent;

{176 Anal_A only_cover_parts_of_the_xiphiplastra anteromedially_overlap_onto_hypoplastra;

{177 Inframarginal_A present absent;

{178 Inframarginal_B* 3_or_more 2;

{179 Inframarginal_C* axillar_and_inguinal_not_in_contact axillar_and_inguinal_in_contact;

{180 Cervical_rib_A present absent;

{181 Cervical_vertebra_A position_of_transverse_processes_middle_of_the_centrum anterior_end_of_the_centrum;

{182 Cervical_vertebra_B ventral_keels_absent_or_slightly_developed_in_all_vertebrae ventral_keels_more_developed_on_posterior_vertebrae;

{183 Cervical_vertebra_C cervical_centrum_8<7_absent present;

{184 Cervical_vertebra_D* triangular_diapophyses_absent triangular_diapophyses_present;

{185 Cervical_articulation_A not_formed formed;

{186 Cervical_articulation_H 8(dorsal 8)dorsal none,_vertebrae_only_meet_at_zygapophyses;

{187 Cervical_vertebra_E* Biconvex_cervical_vertebra_in_the_middle_of_the_neck_absent present;

{188 Cervical_vertebra_F* Biconvex_cervical_vertebra_in_the_middle_of_the_neck_2nd 3rd 4th 5th;

{189 Cervical_vertebra_G* Biconcave_cervical_vertebra_absent present;

{190 Cervical_articulation_I* double_articulation_between_5th_and_6th_absent present;

{191 Cervical_articulation_J* double_articulation_between_6th_and_7th_absent present;

{192 Cervical_articulation_K* Central_articulation_cervical_6-7_concave-convex platicoelous;

{193 Cervical_articulation_L* double_articulation_between_7th_and_8th_absent present;

{194 Cervical_vertebra_H* total_height_of_centra_and_neural_arch_longer_than_the_anteroposterior_length_of_the_cervical_centra_ total_height_of_centra_and_neural_arch_much_shorter_than_the_anteroposterior_length_of_the_cervical_centra_;

{195 Cervical_vertebra_I* neural_arch_on_8th_cervical_not_modified neural_arch_on_8th_cervical_modified_with_the_postzygapophyses_articular_surface_greatly_expanded_AND/OR_pointing_posteroventrally_;

{196 Cervical_vertebra_J* postzygapophyses_not_united_in_midline postzygapophyses_united_in_midline;

{197 Cervical_vertebra_K* Ventral_process_on_cervical_8_absent present_well_developed_(as_tall_or_taller_than_the_high_of_the_centrum);

{198 Dorsal_rib_A length_first_thoracic_rib_long,_extends_full_legth_of_first_costal_and_may_contact_peripherals intermediate,_in_contact_with_axillary_buttresses intermediate_to_short;

{199 Dorsal_rib_B contact_dorsal_rib_9-10_with_costals_present absent;

{200 Dorsal_rib_C dorsal_rib_X_long,_contacting_peripherals dorsal_rib_X_short;

{201 Dorsal_vertebra_A anterior_articulation_of_first_dorsal_centrum_faces_at_most_slightly_anteroventrally faces_strongly_anteroventrally;

{202 Caudal_A tail_club_present absent;

{203 Caudal_B all_centra_amphicoelous formed_centra;

{204 Caudal_C* anterior_caudal_vertebrae_amphicoelous anterior_caudal_vertebrae_procoelous_or_platycoelous anterior_caudal_vertebrae_opisthocoelous;

{205 Caudal_D* posterior_caudal_vertebrae_amphicoelous posterior_caudal_vertebrae_procoelous_or_platycoelous posterior_caudal_vertebrae_opisthocoelous;

{206 Chevron_A present_on_nearly_all_caudals absent_or_poorly_developed_along_posterior_caudals;

{207 Tail_ring_A* absent present;

{208 Tail_ring_B* closed_ventrally open_ventrally;

{209 Tail_club_A* with_three_spikes with_two_pairs_of_spikes_;

{210 Pectoral_girdle_A horizontal_plate_with_a_dorsal_process,_not_triradiate trirradiate;

{211 Pectoral_girdle_B pectoral_girdle_outside_rib_cage pectoral_girdle_inside_rib_cage;

{212 Cleithrum_A present_and_in_contact_with_the_carapace present,_osseous_contact_with_carapace_absent absent;

{213 Scapula_A* lamina_between_the_dorsal_process_of_the_scapula_and_the_acromion_well_developed lamina_between_the_dorsal_process_of_the_scapula_and_the_acromion_reduced:_Kallokibotion lamina_between_the_dorsal_process_of_the_scapula_and_the_acromion_abset;

{214 Humerus_A* Ectepicondylar_foramen_in_a_channel only_a_groove;

{215 Humerus_B* shoulder_present shoulder_absent:_pleurodires;

{216 Humerus_C* lateral_process_in_the_proximal_end_of_the_humerus displaced_from_the_proximal_end,_located_in_the_shaft_of_the_humerus;

{217 Humerus_D* lateral_process_seen_in_dorsal_view lateral_process_not_seen_in_dorsal_view;

{218 Humerus_E* length_of_the_humerus_two_times_or_less_than_the_width_of_the_proximal_end length_of_the_humerus_more_than_two_times_the_width_of_the_proximal_end;

{219 Pelvis_A pelvis-shell_attachment_by_ligaments ischium_attached_to_plastron_by_a_broad_suture ischium_attached_to_plastron_by_its_medial_surface;

{220 Pelvis_B* Thyroid_fenestra_coalescent two_separated_fenestra_completely_or_partially_separated;

{221 Pubis_A* lateral_process_small,_poorly_developed,_columnar lateral_process_well_developed_and_flat;

{222 Pubis_B* Epipubis_process_osseous_or_calcified cartilaginous_or_absent;

{223 Ilium_A elongated_iliac_neck_absent present;

{224 Ilium_B iliac_scar_extends_from_costals_onto_the_peripherals_and_pygal positioned_on_costals_only;

{225 Ilium_C shape_of_articular_site_narrow_and_pointed_posteriorly oval;

{226 Ilium_D posterior_notch_in_acetabulum_absent present;

{227 Illium_E* thelial_process_absent present;

{228 Ischium_A* with_lateral_processes_absent with_lateral_processes_present;

{229 Hypoischium_A present absent;

{230 Manus_A most_digits_with_two_shortened_phalanges most_digits_with_three_elongate_phalanges;

{231 Manus_B paddles_absent short_paddles_present elongate_paddles_present;

{232 Manus_C flippers_absent short_flippers_present elongate_flippers_present;

{233 Pes_A claw_on_5th_digit_present absent;

{234 Pes_B metatarsal_V_functions_as_true_metatarsal metatarsal_V_functions_as_a_tarsal;

{235 Pes_C* 5_digits 4_digits;

{236 Manus_and_Pes_A* carpal_and_tarsal_elements_not_flattened flattened;

{237 Manus_and_Pes_B* Hyperphalangy_manus_digits_4_and_5,_pes_digit_4_no yes;

{238 Posterior_plastral_fontanella posterior_plastral_fontanella_between_the_xiphiplastra_and/or_the_hypoplastra:__absent_in_adult_stage retained_in_adults;

{239 Neural_number less_than_9_elements 9_elements;

{240 Plastron_lobe posterior_lobe_of_plastron__relatively_wide_and_short_ posterior_lobe_of_plastron_elongated_and_narrow_coupled_with_widely_spaced_plastral_buttresses._;

{241 Shape_of_costal_3 costal_3_tapering_towards_the_lateral_side_of_the_shell_or_with_parallel_anterior_and_posterior_borders costal_3_broadens_towards_the_lateral_side_of_the_shell;

{242 Costal_rib distal_portion_of_costal_ribs_not_visible_within_the_costal distal_portion_of_costal_rib_visible_on_the_surface_of_the_costal;

{243 Carapacial_sutures carapacial_elements_finely_sutured_or_the_contact_is_smooth carapacial_sutures_strongly_serrated_in_adult_stage;

{244 First_vertebral vertebral_1_does_not_enter_anterior_margin_of_carapace enters_anterior_margin;

{245 Peripheral_gutter peripheral_gutter_absent_or_only_anteriorly_developed peripheral_gutter_extensively_developed_along_anterior_and_bridge_peripherals;

{246 Costal_rib_distal_end distal_end_of_dorsal_rib_not_visible_or_only_within_costo-peripheral_fontanelles_on_the_dorsal_face_of_the_carapace costo-peripheral_fontanelles_absent,_distal_end_of_posterior_dorsal_ribs_visible_or_distal_end_of_posterior_costals_narrow_and_surrounded_by_the_peripheral;

{247 Tail_length long short;

{248 Cruciform_plastron absent present;

{249 Articulation_of_posterior_cervical_centra circular_or_subcircular_outline greatly_flattened_outline;

{250 Nuchal_emargination absent_or_indistinct present,_excludes_peripheral_1 deep_and_involves_peripheral_1;

{251 Nuchal_posterior_edge less_than_3_times_longer_than_the_lateral_edge more_than_3_times_longer;

{252 Carotid_canal_entry fpcci_is_not_at_back_of_skull fpcci_located_at_back_of_skull_in_pterygoid;

{253 Pterygoid_extension _pterygoid_not_extending_to_posterior_end_of_skull_and_covering_prootic pterygoid_extending_to_posterior_end_of_skull_and_covering_prootic;

{254 Carotid_canal_split not_enclosed_in_bone not_enclosed_but_carotid_canal_is_covered_ventrally_from_the_posterior_end_of_the_skull enclosed_but_carotid_canal_is_not_covered_ventrally_from_posterior_edge_of_skull enclosed_and_carotid_canal_covered_ventrally_from_posterior_edge_of_skull;

;

ccode + 6 18 26 39 41 49 51 58 80 81 88 97 119 120 124 129 132 137 157 158 160 175 212 213 231 232 250 * ] 37 69 70 76 95 96 98 184 197 243;

proc /;

comments 194

{3 67 0 ;

{26 215 preguntarle a Marce!;

{39 37 duda!;

{43 100 absent in Brinkman and Nicholls 2000, present in NMMNH P 57874 (Neurankylus sp);

{44 100 Gaffney 1982;

{45 100 Gaffney 1992, pero P. putorius PU 20600 tiene escamas marcadas, diferentes a escudos...;

{47 100 Baena riparia (USNM ;

{48 100 PU 23662;

{49 100 gaffney 1976;

{50 100 NMS 9145 (SM 594);

{51 100 Gaffney 1975;

{52 15 Rieppel 1980;

{54 18 Kear and Lee (2006);

{54 33 1 in Kear and Lee (2006);

{54 37 Kear and Lee (2006);

{54 95 Kear and Lee (2006);

{54 100 AMNH 1497;

{54 152 Kear and Lee (2006);

{54 184 1 in Kear and Lee (2006);

{54 193 Kear and Lee (2006);

{54 220 Kear and Lee (2006);

{54 236 Kear and Lee (2006);

{55 9 0 in all the specimens I've seen;

{55 100 ZMB 46511

ZMB 46511

;

{55 195 MR: changed from 0 to 1, this was obviously an error by Sterli and de la Fuente in press;

{56 18 Kear and Lee (2006);

{56 37 Kear and Lee (2006);

{56 54 0 in Kear and Lee (2006);

{56 95 Kear and Lee (2006);

{56 100 ZMB 46557

;

{56 152 Kear and Lee (2006);

{56 195 MR: changed from 0 to 1, this was obviously an error by Sterli and de la Fuente in press;

{56 236 Kear and Lee (2006);

{57 18 Kear and Lee (2006);

{57 37 Kear and Lee (2006);

{57 54 1 in Kear and Lee (2006);

{57 95 Kear and Lee (2006);

{57 152 Kear and Lee (2006);

{57 184 1 in Kear and Lee (2006);

{57 236 Kear and Lee (2006);

{58 18 Kear and Lee (2006);

{58 35 1 in Kear and Lee (2006);

{58 37 Kear and Lee (2006);

{58 95 Kear and Lee (2006);

{58 100 MNHN Pal 1870-101

;

{58 152 Kear and Lee (2006);

{58 236 Kear and Lee (2006);

{60 100 USNM 266207

;

{62 177 no veo los inframrginales en el material de MNHN 1870-465;

{63 82 en el especimen del MNHN se abserva que el rostro basisphenoidal es redondo y espeso CAMBI?!!!;

{63 100 MNHN Pal s/n

;

{63 177 no veo los inframarginales... en el material del MNHN...;

{64 100 Sukhanov 2000;

{65 100 USNM 61059

;

{69 16 not visible;

{70 100 USNM 63078

;

{73 10 avevriguar si est? fusionado;

{75 10 check;

{75 100 Meylan and Gaffney (1989);

{77 100 USNM 66666

;

{78 170 checkear! ;

{78 214 Hutchison (1991);

{79 100 MNHN 1991-4543

;

{79 157 Hutchison (1991);

{79 177 me parece que hay un solo par...

;

{79 200 cambi? de 1-->-;

{80 100 USNM 35422

;

{80 147 cambi? de 1-->-;

{80 148 cambi? de 1-->-;

{80 149 cambi? de 1-->-;

{80 157 Hutchison (1991);

{80 177 me parece que hay un solo par...

;

{80 200 cambi? de 1-->-;

{81 16 1 seg?n Meylan and Gaffney (1989);

{81 100 USNM 100554

;

{81 147 cambi? de 1-->-;

{81 148 cambi? de 1-->-;

{81 149 cambi? de 1-->-;

{81 157 Hutchison (1991);

{81 160 Iverson (1991);

{81 179 0 en Iverson (1991), pero lo considera contacto peque?o;

{81 200 cambi? de 1-->-;

{82 16 Meylan and Gaffney (1989);

{82 100 Meylan and Gaffney (1989);

{82 122 Sukhanov et al (2008);

{82 141 0 dice Danilov and Parham (2008);

{82 227 Hirayama (2001); Joyce and Norell (2005);

{84 16 Meylan and Gaffney (1989);

{84 100 Meylan and Gaffney (1989);

{84 141 MyG89;

{84 227 Hirayama (2001); Joyce and Norell (2005);

{85 16 Meylan and Gaffney (1989);

{85 157 Hutchison (1991);

{85 214 Hutchison (1991);

{86 100 USNM 26426

;

{86 237 Delfino et al 2010;

{88 237 Delfino et al 2010;

{89 100 USNM 293690

;

{89 237 Delfino et al 2010;

{91 10 check

;

{91 16 Meylan and Gaffney (1989);

{91 100 Meylan and Gaffney (1989)

;

{92 237 Delfino et al 2010;

{93 31 MR: this was mistakenly scored as 0 in Rabi et al. (2013). This is of course 1 (united).;

{93 59 reduced interpterygoid vacuity also present;

{93 93 MR: Rabi 2013 missunderstood this character, this should be 0 not 2.;

{94 31 MR: this was mistakenly scored as 0 in Rabi et al. (2013). This is of course 1 (united).;

{95 31 MR: this was mistakenly scored as 0 in Rabi et al. (2013). This is of course 1 (united).;

{96 31 MR: this was mistakenly scored as 0 in Rabi et al. (2013). This is of course 1 (united).;

{96 64 a contact is absent according to Brinkman et al. 2013 but it has to be confirmed;

{96 86 MR: changed it from 1 to 0 (it was a mistake by Rabi et al. 2013);

{96 93 MR: Rabi 2013 missunderstood this character, this should be 0 not 2.;

{97 184 MR: this is changed from 1 to ? contra Rabi et al. 2013 (it was a mistake);

{97 200 MR:This is seen on the left side of IVPP V9537-1 (contra Brinkman and Peng (1993) ;

{99 17 MR: changed from ? to 1;

{99 37 MR: Changed from 1 to 0 after personal observation of holotype;

{99 39 MR: changed this from ? to 0 after pers. obs. of holotype ;

{99 56 MR: pers. obs. of holotype;

{99 61 MR: contact is barely present in some, barely absent in other specimens;

{99 64 MR: this was scored as 1 and I changed it to 0 (obviosuly it was a mistake);

{99 75 MR: changed it from 1 to 0 after pers. obs. of holotype;

{99 82 RM: after material at PIN;

{99 85 MR: absent after pers obs of holotype;

{99 91 MR: after pers. obs. of PIN material: ? to 0;

{99 118 MR: after PIN material;

{99 126 MR: this is changed from ? to 0 after pers.obs. of PIN material. ;

{99 128 MR: this is changed from ? to 1.;

{99 141 MR: after PIN material;

{99 152 MR: changed from 0 to 1 after revision of PIN material. ;

{99 157 MR: after PIN material;

{99 180 MR: this character is ambigous based on photos obtained from V Sukhanov. I changed it from ? to 0. ;

{99 182 MR: changed it from 0 to 1 because it is similar to the extent seen in Dracohelys which was scored 1 by Sterli and de la Fuente (in press);

{99 183 MR: after photos from V Sukhanov;

{99 194 MR: this is changed from ? to 1 based on photos from V. Sukhanov ;

{99 195 MR: changed from ? to 1 based on photos from V Sukhanov;

{99 196 MR: changed it from ? to 0 based on photos from Sukhanov;

{99 203 MR: based on PIN material;

{99 210 MR: after PIN material;

{99 212 MR: after PIN material;

{99 213 MR: after PIN material;

{99 216 MR: after PIN material;

{99 217 MR: after PIN material;

{99 218 MR: after PIN material;

{99 223 MR: after PIN material;

{100 16 it does not say

;

{100 67 MR: changed it from ? to 1 after Gaffney et al. 2007;

{100 91 MR: after pictures of TMP material: ? to 1;

{100 100 MR: pictures of TMP material reveal that the specimen is not well preserved enough to score this character;

{100 122 MR: after TMP material;

{100 183 MR: after Parham and Hutchison 2003;

{100 212 MR: after photos of TMP material;

{101 250 this contrasts with the reconstruction of Brinkman et al. (2013) but unpublished specimens from the same collection reveal the emargination is not extending to the peripheral ;

{102 3 MR: This is changed from 1 to ? because the specimen here is really damaged and the prefrontals can not be discerned with confidence. ;

{102 6 MR: This is changed from 1 to ? because the outline of the prefrontals are unclear in the holotype (and only specimen). ;

{102 7 MR: This is changed from 1 to ? because the outline of the prefrontals are unclear in the holotype (and only specimen). ;

{102 11 MR: This is changed from 1 to ? because the holotype (and only specimen) is damaged here. ;

{102 16 MR: This is changed from ? to 0 after personal observation of the type specimen. ;

{102 17 MR: changed from ? to 1;

{102 18 MR: changed from 2 to ? because the rim of the temporal region is incomplete all along. ;

{102 19 MR: changed from 1 to ? because this region is not preserved. ;

{102 71 MR: changed it from 1 to ? because the region is incomplete;

{102 83 MR: absent after pers. obs. of holotype;

{102 136 MR: after Tong and Brinkman (2013) and personal observation;

{102 150 MR: based on the outline of the hyoplastra as illustrated in Brinkman 2001;

{102 196 MR: based on Tong and Brinkman who does not say that it is any different in this regard from Sinemys brevispinus. ;

{102 233 MR: I changed this from - to ?;

{104 16 not preserved

;

{104 18 MR: changed from 2 to 1 because it is similar to the less emarginated condition of Hangaiemys. ;

{104 19 MR: changed from ? to 1 after Brinkman and Wu (1999);

{104 35 MR: changed from ? to 0 after Brinkman and Peng (1999);

{104 36 MR: changed from ? to 0 after Brinkman and Peng (1999);

{104 38 MR: changed from ? to - after Brinkman and Peng (1999);

{104 39 MR: changed from ? to 0 after Brinkman and Peng (1999) and after Brinkman and Peng (1993);

{104 45 MR: after Brinkman and Wu 1999;

{104 46 MR: after Brinkman and Wu 1999;

{104 54 Although Brinkman and Wu (1999) clains that the pto is largely formed by the prootic we are unsure what their definition of the pto is and since confusion is often arising with this structure we leave it as unknown (as Sterli and de la Fuente in press, though they just probably missed to score this character);

{104 55 MR: after Brinkman and Wu (1999);

{104 67 MR: changed from ? to 1 after Brinkman and Wu (1999);

{104 71 changed from 1 to ? because it is too incomplete;

{104 77 MR: after Brinkman and Wu 1999;

{105 83 MR: present after pers. obs. of holotype, faint but present. contra Tong and Brinkman 2013;

{106 21 MR: changed it from 0 to ?: "Neither specimen preserves a quadratojugal": Brinkman and Peng (1993);

{106 50 MR: changed it from 0 to ? because this region is not preserved. ;

{106 52 MR: not preserved, I canged it from 2 to ?;

{106 54 MR: changed from 1 to 0 after Brinkman and Peng 1993;

{106 74 MR: not visible. ;

{106 99 MR: changed from 0 to ?;

{106 100 Brinkman and Peng 1993;

{106 153 MR: I changed it from - to 0 to reflect the distinct morphology of Sinemys spp. where the epi and entoplastra are not narrow eventhough they are also different from the squarish condition of most other turtles. ;

{106 189 MR: changed from 0 to ?, not preserved/prepared;

{107 0 inferred from the anterior outline of the prefrontal and the frontal;

{107 24 MR: contact not actually preserved but inferred from the posteriorly extending postorbital. ;

{110 3 MR: prefrontals are not preserve in situ, but the articulation surfaces with the frontals show that the prefrontals must have had a medial contact ;

{111 93 MR: Rabi 2013 missunderstood this character, this should be 0 not 2.;

;
